# Genetic Variability of 27 Traits in a Core Collection of Flax (*Linum usitatissimum* L.)

**DOI:** 10.3389/fpls.2017.01636

**Published:** 2017-09-21

**Authors:** Frank M. You, Gaofeng Jia, Jin Xiao, Scott D. Duguid, Khalid Y. Rashid, Helen M. Booker, Sylvie Cloutier

**Affiliations:** ^1^Morden Research and Development Centre, Agriculture and Agri-Food Canada Morden, MB, Canada; ^2^Crop Development Centre, Department of Plant Sciences, University of Saskatchewan Saskatoon, SK, Canada; ^3^Department of Agronomy, Nanjing Agricultural University Nanjing, China; ^4^Ottawa Research and Development Centre, Agriculture and Agri-Food Canada Ottawa, ON, Canada

**Keywords:** phenotypic and genetic variability, agronomic traits, fiber, seed quality, fatty acid composition, core collection, linseed, flax

## Abstract

Assessment of genetic variability of plant core germplasm is needed for efficient germplasm utilization in breeding improvement. A total of 391 accessions of a flax core collection, which preserves the variation present in the world collection of 3,378 accessions maintained by Plant Gene Resources of Canada (PGRC) and represents a broad range of geographical origins, different improvement statuses and two morphotypes, was evaluated in field trials in up to 8 year-location environments for 10 agronomic, eight seed quality, six fiber and three disease resistance traits. The large phenotypic variation in this subset was explained by morphotypes (22%), geographical origins (11%), and other variance components (67%). Both divergence and similarity between two basic morphotypes, namely oil or linseed and fiber types, were observed, whereby linseed accessions had greater thousand seed weight, seeds m^−2^, oil content, branching capability and resistance to powdery mildew while fiber accessions had greater straw weight, plant height, protein content and resistance to pasmo and fusarium wilt diseases, but they had similar performance in many traits and some of them shared common characteristics of fiber and linseed types. Weak geographical patterns within either fiber or linseed accessions were confirmed, but specific trait performance was identified in East Asia for fiber type, and South Asia and North America for linseed type. Relatively high broad-sense heritability was obtained for seed quality traits, followed by agronomic traits and resistance to powdery mildew and fusarium wilt. Diverse phenotypic and genetic variability in the flax core collection constitutes a useful resource for breeding.

## Introduction

Flax (*Linum usitatissimum* L.) is a multipurpose crop grown for production of stem fiber and seed oil (Singh et al., 2011). Due to long-term domestication for fulfillment of these purposes, cultivated flax has diversified into two main types, namely fiber and oil or linseed types, as well as an intermediate type (Liu et al., 2011). These types differ considerably in morphology, growth habits and agronomic traits. Fiber-type plants are usually taller and have fewer branches while linseed types are often shorter, have more branches and produce more seeds (Diederichsen and Ulrich, 2009). Linseed is used for food, feed and industrial applications (Singh et al., 2011). Flax seeds contain digestible proteins and lignans and their oil is rich in health-beneficial omega-3 fatty acid known as alpha linolenic acid (Oomah, 2001). Flax oil can easily oxidize and harden in contact with the air; hence, it can be used in paints, varnishes, inks, putty, linoleum and other industrial applications (Juita et al., 2012). Fiber flax provides fibers for linens, woven or nonwoven textiles, twine and rag-based paper (Deyholos, 2006). Both types can serve as feedstock for the production of biomass energy in the biofuel industry (Naik et al., 2010). Most varieties are either oilseed or fiber types as opposed to dual purpose (Deyholos, 2006) but the intermediate type opens the door for development of a true dual purpose flax (Irvine et al., 2010) where both stems and seeds have commercial outcomes (You et al., 2016b).

Flax thrives best in regions with temperate climates under favorable growing conditions, such as moderate warmth, high moisture and well-drained medium heavy soils (Worku et al., 2015). Currently, flax is primarily cultivated in western Canada (linseed), the cool-temperate and continental regions of China (fiber and linseed), north-central USA (linseed) and Western Europe (fiber) (Foulk et al., 2004; Liu et al., 2011; You et al., 2016b). As of 2011, flax is the third largest textile fiber crop and fifth largest oil crop in the world and, Canada is the world's largest exporter of flax seeds (Worku et al., 2015).

Flax domestication is hypothesized to have occurred during the Neolithic period between 8,000 and 10,000 years ago in the Near-Middle East from where it spread to Europe, the Nile Valley and over the rest of the world (Hillman, 1975; Van Zeist and Bakker-Heeres, 1975). However, modern improvement of flax has lagged behind other oilseed crops, such as soybean and oilseed brassicas, and fiber crops, such as cotton. Germplasm is the basis of plant breeding programs. Since 1910, a total of 82 flax cultivars have been registered by Canadian flax breeding programs, but the genetic base of this germplasm is relatively narrow as indicated by a coefficient of parentage of 0.14 (You et al., 2016b). The introduction of new germplasm is needed to broaden the genetic diversity and invigorate breeding stocks. Presently, the *ex situ* world collections contain approximately 48,000 flax accessions (Diederichsen and Fu, 2008) and, 3,378 of them are housed at Plant Gene Resources of Canada (PGRC). A core collection comprising 381 of these accessions was assembled (Diederichsen et al., 2013). This core subset preserves the variation present in the whole collection and represents a broad range of geographical origins (38 countries), both fiber and linseed types and different improvement statuses such as landraces, breeding lines and cultivars (Diederichsen et al., 2013). A total of 26 additional breeding lines and cultivars from Canadian flax breeding programs have since been added to this core subset to ensure inclusion of relevant modern lines, resulting in a current core collection of 407 flax accessions. This core collection was characterized at the molecular level using 448 microsatellite markers (Soto-Cerda et al., 2013). It was also evaluated in field trials from 2009 to 2012 under the Total Utilization Flax Genomics (TUFGEN) project, for a total of 27 traits including agronomic, seed quality, fiber and disease resistance traits. The objectives of the present study were to comprehensively characterize phenotypic and genetic variabilities of these traits within the core collection and their associations based on morphotypes and geographical origins of the core collection. The assessment of genetic variability for the core collection would constitute a useful resource and guidance for better germplasm utilization in flax genetic improvement.

## Materials and methods

### Flax accessions from the core collection

The flax core collection contains a total of 407 accessions. However, 391 out of 407 accessions accommodated the field layout design described below, and thus 16 Canadian flax cultivars were excluded for field trials. These 391 accessions consisted of 20 landraces, 90 breeding lines, 245 varieties from different breeding programs and 36 accessions of unknown improvement status. These comprised 273 linseed, 89 fiber and 29 unknown types from 38 countries. To facilitate analysis, the geographical origins of the accessions were divided into 11 subgroups: North America (NA), South America (SA), Eastern Asia (EA), Western Asia (WA), Southern Asia (SA), Central and Eastern Europe (CEE), Western Europe (WE), Southern Europe (SE), Northern Europe (NE), Oceania (OC), and Africa (AF). Detailed information of the accessions is provided in Tables S1 and S2.

### Field experimental design

The 391 accessions were evaluated for agronomic, seed quality and fiber traits in field trials from 2009 to 2012 at two Canadian locations: Morden, Manitoba and Kernen Crop Research Farm near Saskatoon, Saskatchewan. Evaluation of resistance to diseases was conducted from 2010 to 2015 at Morden, Manitoba. A type-2 modified augmented design (MAD2) (Lin and Poushinsky, 1985) was used for the field trials from which phenotypic data were collected. The field layout was designed to have 100 whole plots arranged in a 10 row by 10 column grid (You et al., 2013). Each 2 × 2 m whole plot was split into five subplots. The 391 accessions represented one control accession and 390 test accessions. The main plot control cultivar “CDC Bethune” was placed in the center subplot of each whole plot. Cultivars “Macbeth” and “Hanley,” the subplot controls, were randomly assigned to any of the four remaining subplots of each of five randomly selected whole plots. The remaining 390 test accessions were then randomly assigned to the remaining 390 subplots. Thus, this design contained a total of 500 subplots, accommodating one control accession in the 100 central subplots plus 390 test accessions in the remaining 400 subplots. The design and assignment of test accessions were performed using Agrobase (Agronomix Software Inc, Winnipeg, MB, Canada). This experimental design was consistently used for all trials regardless of years and locations without substitution of any test lines as previously described (You et al., 2013).

### Phenotyping of 27 traits

Ten agronomic, eight seed quality, six fiber and three disease resistance traits, for a total of 27 traits, were evaluated (Table 1). Plant height (PLH) was measured from the ground to the uppermost plant part at boll maturity. Days to flowering (DTF) were recorded as the number of days from sowing to 95% flowering, and days to maturity (DTM) from sowing to 95% brown bolls, i.e., when seeds rattled in the bolls. Branching score (BSC), which represents the branching architecture, was determined as previously described (Diederichsen and Richards, 2003), with 1 = 1/1, 2 = 1/2, 3 = 1/3, 4 = 1/4, 5 = 1/5, and 6 = 1/6 of the total stem length branched from the top. Generally, a higher branching score means a smaller number of branches on the main stem and less branching capability because branches are restricted to a smaller area. Lodging (LOD) was recorded at maturity on a scale of 1–9, where a score of 1 represents upright plants. Seed yield (YLD) was calculated from the seeds harvested from 2 × 0.5 m row sections located in the central part of each subplot. Yield components and other agronomic traits such as thousand-seed weight (TSW), seeds boll^−1^ (SEB), bolls m^−2^ (BM2), and seeds m^−2^ (SM2), were determined as previously described (Soto-Cerda et al., 2014).

**Table 1:** Phenotypic performance and estimates of genetic parameters of 27 agronomic, seed quality, fiber and disease resistance traits in the flax core collection.

**Trait group**	**Trait**	**Abbreviation**	**$\bar{x}$ ± s**	**Range**	**$\hat{CV}$(%)**	**$\hat{GCV}$**	***ΔG* (%)**	**${\hat{H}}^{2}$**
Agronomic	Seed yield (t·ha^−1^)	YLD	0.78 ± 0.27	0.07 – 1.46	34.15	17.90	23.61	0.41
	Seeds boll^−1^	SEB	6.10 ± 0.88	2.80 – 8.12	14.47	12.23	14.03	0.31
	Seeds m^−2^	SM2	10,804 ± 3,039	2,638 – 27,714	28.13	23.45	28.58	0.35
	Thousand-seed weight (g)	TSW	5.36 ± 0.86	2.90 – 8.42	16.01 (20.9)^#^	14.01	25.33	0.77
	Bolls m^−2^	BM2	1,763.33 ± 386.55	736.32 – 3,821	21.92	17.22	20.07	0.32
	Lodging	LOD	1.36 ± 0.40	0.87 – 3.32	29.20	25.74	19.84	0.14
	Days to flowering	DTF	51.13 ± 3.07	45.53 – 70.74	6.00 (8.1)	5.11	8.55	0.66
	Days to maturity	DTM	97.40 ± 3.85	88.06 – 110.7	3.96	3.77	3.56	0.21
	Plant height (cm)	PLH	51.15 ± 12.81	23.00 – 95.49	25.04 (27.8)	24.63	38.97	0.59
	Branching score	BSC	3.50 ± 1.41	1.00 – 6.00	40.28	38.85	19.60	–
Seed quality	Protein content (%)	PRO	26.88 ± 1.80	16.73 – 31.3	6.71	5.30	9.20	0.72
	Oil content (%)	OIL	42.13 ± 1.88	37.22 – 50.59	14.46 (4.6)	13.17	18.99	0.64
	Iodine value	IOD	186.10 ± 7.02	145.41 – 202.85	3.77	3.70	6.82	0.80
	Palmitic (%)	PAL	5.48 ± 0.60	3.30 – 8.45	11.01 (12.7)	10.92	20.37	0.82
	Stearic (%)	STE	4.33 ± 1.01	2.33 – 9.4	23.37 (26.6)	21.67	41.16	0.85
	Oleic (%)	OLE	21.21 ± 3.03	13.82 – 37.97	14.30 (14.5)	13.13	23.73	0.77
	Linoleic (%)	LIO	14.22 ± 4.15	6.84 – 68.43	29.18 (15.0)	29.01	57.63	0.93
	Linolenic (%)	LIN	54.75 ± 4.70	5.02 – 66.07	8.59 (7.5)	8.32	13.92	0.66
Fiber	Straw weight (g)	STR	23.80 ± 12.56	4.58 – 75.01	52.76	39.58	65.74	0.65
	Fiber (%)	FIB	38.50 ± 1.99	34.47 – 46.52	5.16 (16.0)	5.14	6.08	0.33
	Lignin (%)	LIG	9.48 ± 0.29	8.32 – 10.08	3.03	3.02	3.52	0.32
	Shive (%)	SHI	62.08 ± 2.01	53.98 – 66.11	3.23	3.11	3.68	0.33
	Cell walls (%)	CEW	79.17 ± 0.96	75.86 – 82.01	1.21	1.07	0.91	0.17
	Cellulose (%)	CEL	60.14 ± 1.99	54.21 – 65.21	3.31	3.22	3.69	0.31
Disease resistance	Pasmo score	PAS	3.40 ± 0.96	1.35 – 7.15	28.09	24.24	24.97	0.25
	Powdery mildew score	MIL	4.13 ± 1.46	1.36 – 8.45	35.29	29.49	43.81	0.52
	Fusarium wilt score	WIL	7.77 ± 1.37	4.00 – 10.1	17.64	13.71	21.87	0.60

*$\bar{x}$, population mean; s, standard deviation; $\hat{CV}$, coefficient of variation; $\hat{GCV}$, genetic coefficient of variation; ΔG, expected genetic advance at 5% of the selection intensity; ${\hat{H}}^{2}$, broad-sense heritability*.

# *CV values in parentheses are from (Diederichsen et al., 2013) and represent the estimated variation of the whole collection of 3,378 accessions. Heritability of BSC could not be obtained because of insufficient environments*.

A total of 1 g of seed from each accession from each environment was sampled for measurement of protein content (PRO), oil content (OIL), and fatty acid composition (FAC). FAC includes palmitic acid (PAL), stearic acid (STE), oleic acid (OLE), linoleic acid (LIO), and linolenic acid (LIN). FAC for all test accessions was obtained by gas chromatography (Varian 3800, Varian Analytical Instruments, Mississauga, ON, Canada) of fatty acids methyl esters extracted from seeds according to AOAC method 996.06 (Daun et al., 1983; Association of Official Analytical Chemists, 2001) and IOD, an indicator of the degree of unsaturation, was calculated (Cloutier et al., 2010). OIL was determined by nuclear magnetic resonance (NMR) spectroscopy calibrated against the FOSFA extraction reference method. The protein content was measured using near-infrared (NIR) spectroscopy calibrated against the combustion analysis reference method and expressed on an N × 6.25 dry basis. Phenotyping of these seed quality traits has been previously described (Soto-Cerda et al., 2014).

Fiber traits, including percent fibers (FIB), cell walls (CEW), cellulose (CEL), shive (SHI) and lignin (LIG), were determined by NIR spectroscopy and a calibration curve developed by Light Solutions (Alpharetta, Georgia, USA) and Schweitzer Mauduit (Winkler, Manitoba, Canada) was provided to us by the Composite Innovation Center (Winnipeg, Manitoba, Canada). Straw weight (STR) was measured based on the fresh weight of the straw of 2 × 0.5 m rows after boll stripping.

Disease reactions to fusarium wilt (WIL) caused by the fungus *Fusarium oxysporum* f. sp. *lini* (Bolley) Snyd. & Hans, pasmo (PAS) cause by the fungus *Septoria linicola* (Speg) Garassini (sexual state *Mycosphaerella linorum* Naumov) and powdery mildew (MIL) caused by *Oidium lini* Skoric, were independently evaluated in separate disease nurseries at Morden, MB from 2010 to 2015.

For fusarium wilt evaluation, the trials relied on natural infection in the wilt nursery where susceptible cultivars have been continually seeded since 1950. The flax cultivars Bison and Novelty served as resistant and susceptible checks, respectively, and were seeded after every 10 flax entries. The same experimental design described above was adopted. Disease assessment was conducted at seedling, early flowering and late flowering/green boll stages using a 0–9 scale where 0 represents vigorous plants devoid of any signs of wilt and 9 corresponds to plots where all plants were severely wilted or dead (Rashid and Kenaschuk, 1993). An overall score for each accession was obtained by averaging the ratings across the three stages.

For pasmo evaluation, the infested straw from the previous growing season was used as source of inoculum. Each accession was seeded in 3 m rows with 30 cm row spacing during the 2nd to 3rd week of May every year. Approximately 200 g of infested chopped straw were spread between rows at the early growing stage when plants were approximately 30 cm tall. A misting system was operated for 5 min every half hour for 4 weeks, except on rainy days, to help spread conidia from infected stubble and to ensure disease infection and development. Disease was assessed weekly on leaves and stems using a 0–9 scale where 0 means no sign of disease and 9 means the majority of leaves or stems were infected. Average scores of all ratings were used to represent the disease reaction.

For powdery mildew evaluation, pathogen infected plants from the greenhouse were transplanted into the field at the early flowering stage to ensure early disease infection and development in the field. One pot containing ten infected plants was transplanted every ten rows. Each flax entry was seeded in 3m rows spaced 30 cm apart during the 2nd to 3rd week of May every year. Disease ratings on leaves and stems were conducted weekly using a 0–9 scale where 0 means no sign of powdery mildew infection and 9 means that most of the leaves were infected (Rashid and Duguid, 2005). Average scores were used to represent the disease reaction for each accession.

For all three diseases, a score of 0–2 was considered resistant (R), 3–4, moderately resistant (MR), 5–6, moderately susceptible (MS) and 7–9, susceptible (S) phenotypes.

### Analysis of variance and genetic parameter estimation

All phenotypic data from the field trials and laboratory measurements were adjusted as previously described using the MAD pipeline (You et al., 2013). The adjusted phenotypic data were analyzed using a linear model:

(1) $$\begin{array}{r} y_{ijk}=\mu +G_{i}+Y_{j}+{\left(GY\right)}_{ij}\;+\;S_{k}\;+\;{\left(GS\right)}_{ik}\;+\;{\left(YS\right)}_{jk}\\ +\;{\left(GYS\right)}_{ijk}\;+\;\varepsilon_{ijk} \end{array}$$

(*i* = 1, 2, …, *g, j* = 1, 2, …, *y, k* = 1, 2, …, *s*),

where *y*_*ijk*_ ~ *N*(μ, $\sigma_{P}^{2}$), *G*_*i*_ ~ *N*(0, $\sigma_{G}^{2}$), *Y*_*j*_ ~ *N*(0, $\sigma_{Y}^{2}$), (*GY*)_*ij*_ ~ *N*(0, $\sigma_{GY}^{2}$), *S*_*k*_ ~ *N*(0, $\sigma_{S}^{2}$), (*GS*)_*ik*_ ~ *N*(0, $\sigma_{GS}^{2}$), (*YS*)_*jk*_ ~ *N*(0, $\sigma_{YS}^{2}$), (*GYS*)_*ijk*_ ~ *N*(0, $\sigma_{GYS}^{2}$), and ε_*ijk*_ ~ *N*(0, $\sigma_{e}^{2}$). $\sigma_{P}^{2}$, $\sigma_{G}^{2}$, $\sigma_{Y}^{2}$, $\sigma_{GY}^{2}$, $\sigma_{S}^{2}$ $\sigma_{GS}^{2}$, $\sigma_{YS}^{2}$, $\sigma_{GYS}^{2}$, and $\sigma_{e}^{2}$ are variances for phenotype, genotype (G), year (Y), G × Y, site (S), G × S, Y × S, G × Y × S, and error, respectively. $\sigma_{e}^{2}$ was jointly estimated based on replicated control genotypes during *y* years at *s* sites. Variance and covariance components of genotypes (G), environments (E), and their interactions were estimated using the MAD pipeline (You et al., 2016d).

Broad-sense heritability (*H*^2^) of a trait on a plot basis across environments was used because the entry mean based *H*^2^ was overestimated in the MAD2 design (You et al., 2016a). *H*^2^ was approximated using the inter-environment correlation (*r*_*E*_) method (You et al., 2016c). The coefficients of variation ($\hat{CV}$) and genetic $\hat{CV}$ ($\hat{GCV}$) of traits were estimated as $\hat{CV}$ = ${\hat{\sigma }}_{P}/\bar{x}$ and $\hat{GCV}$ = ${\hat{\sigma }}_{G}/\bar{x}$, respectively, where ${\hat{\sigma }}_{P}$, ${\hat{\sigma }}_{G}$, and $\bar{x}$ are the phenotypic and genetic standard deviations and population mean of a trait, respectively. The expected genetic advance for selection of a trait (Δ*G*, %) based on phenotype was calculated as Δ*G* = *k*${\hat{\sigma }}_{G}\sqrt{{\hat{H}}^{2}}/\bar{x}$ = *k*$\hat{GCV}\sqrt{{\hat{H}}^{2}}$, where *k* is the intensity of selection which would equal 2.06 if 5% of the individuals were selected from the normally distributed population and, where $\bar{x}$ is the population mean of the trait. Variance components of a trait explained by morphotype and geographical origin of accessions were estimated using the SAS VARCOMP procedure (SAS, Cary, USA). For each trait, a random effect model “y = morphotype geographical_region” with the restricted maximum likelihood method (METHOD = REML) was used to estimate variances for morphotype, geographical region and residual. The absolute values of variances were then converted to proportions of the total variance.

### Discriminant, principal component, and cluster analyses

A linear discriminant function of morphotypes was constructed based on the 362 accessions of known morphotype to categorize accessions of unknown morphotype into fiber or linseed types using the SAS DISCRIM procedure with options “METHOD = NORMAL POOL = NO CROSSVALIDATE,” i.e., the normal-theory method (METHOD = NORMAL) assuming unequal variances (POOL = NO) in two morphotypes was used to construct linear discrimination function, and the CROSSVALIDATE option to display cross validation error-rate estimates. The linear discrimination function for morphotype contains coefficients for the constant term and 27 traits (or variables) for fiber and linseed type, respectively. Cross-validation was performed to assess the classification accuracy. Then the discrimination function was applied to each of the 29 accessions of unknown morphotype to calculate posterior probability of membership in the fiber or linseed morphotype groups. According to the posterior probability of an accession in fiber and linseed, the morphotype with a higher probability was assigned to the accession.

Principal component analysis (PCA) and cluster analysis were performed to analyze trait variations. The first several principal components (PCs), accounting for more than 85% of the cumulative variance, were used to calculate Euclidean distances among accessions for fiber and linseed accessions, respectively. The R (v2.5, http://cran.r-project.org/) package “*prcomp*” was used for PCA. The biplot of the first two PCs was drawn using *ggplot* function with a function of *state_ellipse (level* = *0.95)* to draw 95% normal confidence ellipses. The Euclidean distance matrix of accessions was calculated using the “*dist*” function with the “euclidean” method. The Ward algorithm in the function “*hclust*” of the R package “*stats*” was used for hierarchical cluster analysis. The means and standard deviations of traits for clusters were obtained from cluster analysis. A one-way ANOVA with multiple comparisons (Tukey's range test) was performed to test significance among different clusters.

To explore the relationship of trait performance with geographical origin, the means of traits for different geographic regions were calculated and compared using one-way ANOVA with multiple comparisons (Tukey's range test) to test significance among different geographical regions. In addition, the Euclidean distances among accessions were averaged with respect to geographical regions using the function “*meandist*” of the R package “*vegan*” to calculate mean within-region (diagonal) and between-region distances. Then the matrix of between-region distances was further analyzed for cluster analysis. The R package “*ggplot2*” was used to draw figures.

## Results

### Phenotypic and genetic variation

Significant differences among accessions were observed for all 27 traits in both years and locations (Table S3). As expected, the $\hat{GCV}\;$was smaller than the $\hat{\;CV}$ for all traits but close to the$\hat{\;CV}\;$for most traits. Seventeen traits showed large phenotypic and genetic variations, with $\hat{CV}$ and $\hat{GCV}$ values greater than 10% (Table 1). Four traits had a $\hat{CV}$ exceeding 30%, seven ranged from 20 to 30%, six from 10 to 20% and ten less than 10%. Disease resistance and agronomic traits had the largest average $\hat{CV}$ of 27.0 and 19.8%, respectively, while seed quality and fiber traits had similar average $\hat{CV}$ of 13.9 and 11.5%, respectively. STR, an indicator of biomass or fiber yield in fiber accessions, and YLD had the largest $\hat{CV}$ values of 52.8 and 34.2%, respectively. Except for STR, all other fiber traits had very low variation (less than 6%). Expected genetic advance (Δ*G*) showed that high potential selection gains of more than 10% were expected in 18 traits if 5% of the accessions were selected; this was particularly high for STR (65.7%), PLH (39.00%), LIO (57.6%), MIL (43.8%), SM2 (28.6%), and TSW (25.3%).

**Figure 1:**
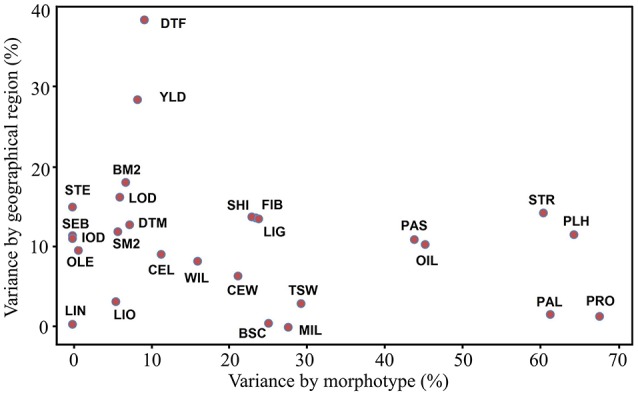
Bi-plot of the phenotypic variation of 27 traits explained by morphotype and geographical origin. The variances explained by morphotype, geographical origin and other factors were standardized to percentages (%) based on the total variance for each trait. BM2, bolls m^−2^; BSC, branching score; CEL, cellulose (%); CEW, cell walls (%); DTF, days to flowering; DTM, days to maturity; FIB, fiber (%); IOD, iodine value; LIG, lignin (%); LIN, linolenic (%); LIO, linoleic (%); LOD, lodging score; MIL, powdery mildew rating; OIL, oil content (%); OLE, oleic (%); PAL, palmitic (%); PAS, pasmo rating; PLH, plant height (cm); PRO, protein content (%); SEB, seeds boll^−1^; SHI, shive (%); SM2, seeds m^−2^; STE, stearic (%); STR, straw weight (g); TSW, thousand-seed weight (g); YLD, seed yield (t·ha^−1^); WIL, fusarium wilt rating.

Phenotypic variations of all accessions were partitioned into components according to their morphotype, geographical origin and other factors for all 27 traits (Table S4). On average, morphotype and geographical origin accounted for 22.0 and 11.0% of the total phenotypic variation, respectively. Most (67.0%) of the total variance was caused by other variation among accessions within morphotype and geographical origin. A total of 13 traits (PRO, PLH, PAL, STR, OIL, PAS, TSW, MIL, BSC, LIG, FIB, SHI, and CEW) contributed to more than 20% of the variation within morphotypes. Within geographical origin, DTF and YLD explained 38.4 and 28.5% of the variation, respectively (Figure 1).

### Broad-sense heritability

The broad-sense heritability of a trait represents the extent with which genotypes are affected by environment and experimental error, a measurement that allows breeders to understand the accuracy or repeatability of phenotypic selection in breeding (You et al., 2016a). Here, we estimated *H*^2^ for 26 of the 27 traits under study (Table 1, BSC was excluded because of insufficient environments). All eight seed quality traits, including PRO, OIL, IOD and the five FACs, had relatively high ${\hat{H}}^{2}$ values, ranging from 0.64 to 0.93. Except for TSW (0.77), PLH (0.59), DTF (0.66) and STR (0.65), relatively low ${\hat{H}}^{2}$ values were calculated for agronomic and fiber traits which averaged 0.38 and 0.35, respectively. For the three disease resistance traits, MIL and WIL had moderate ${\hat{H}}^{2}\;$values of 0.52 and 0.60, respectively, whereas PAS had a low ${\hat{H}}^{2}$ value of 0.25.

### Divergence and similarity between linseed and fiber flax

**Figure 2:**
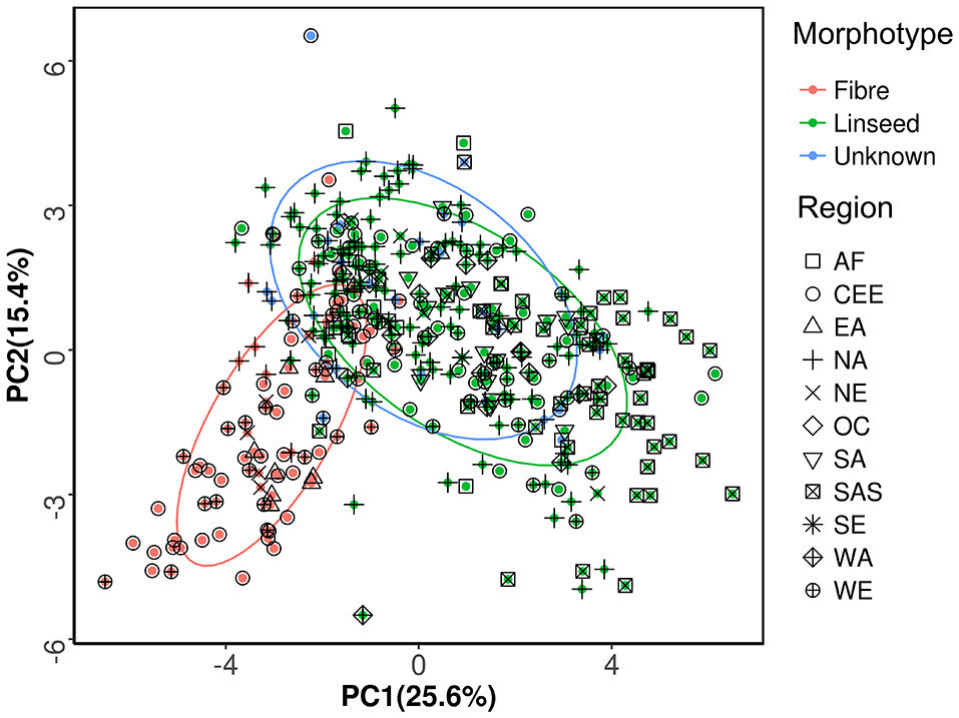
Principal component analysis of the 391 flax accessions of the core collection. The first and second principal components, accounting for 41% of the total variance, are presented. The percentages in parentheses in the axis titles represent the variance explained by each of the two principal components. The ellipses represent the 95% confidence limits of fiber, linseed and unknown morphotypes.

Available information for the 391 accessions indicated that 273 accessions were of linseed type, 89 were of fiber type, and the remaining 29 accessions were of unknown morphotype (Table S1). PCA of the 391 accessions was performed based on the phenotypic data of 27 traits. The bi-plot of the first two principal components (PCs) showed that the fiber and linseed accessions formed two distinct but somewhat overlapping groups (Figure 2). The overlap between the two groups indicated that some accessions have characteristics of both fiber and linseed types. Most of the 29 accessions of unknown type located within the confidence circle of either the fiber or linseed groups. To clarify the morphotype of the accessions of unknown type, discrimination analysis using data of the 27 traits of the 362 accessions of known morphotype was conducted to generate a linear discriminant function (Table S5). High correct discrimination rates of 99.3% for linseed and 95.4% for fiber flax were obtained in cross-validation. This discrimination function was thus applied to discriminate the morphotypes of the 29 unknown accessions which were partitioned into three fiber and 26 linseed types. As a result, the 391 accessions were regrouped into 299 linseed and 92 fiber types (Tables S1, S2).

**Figure 3:**
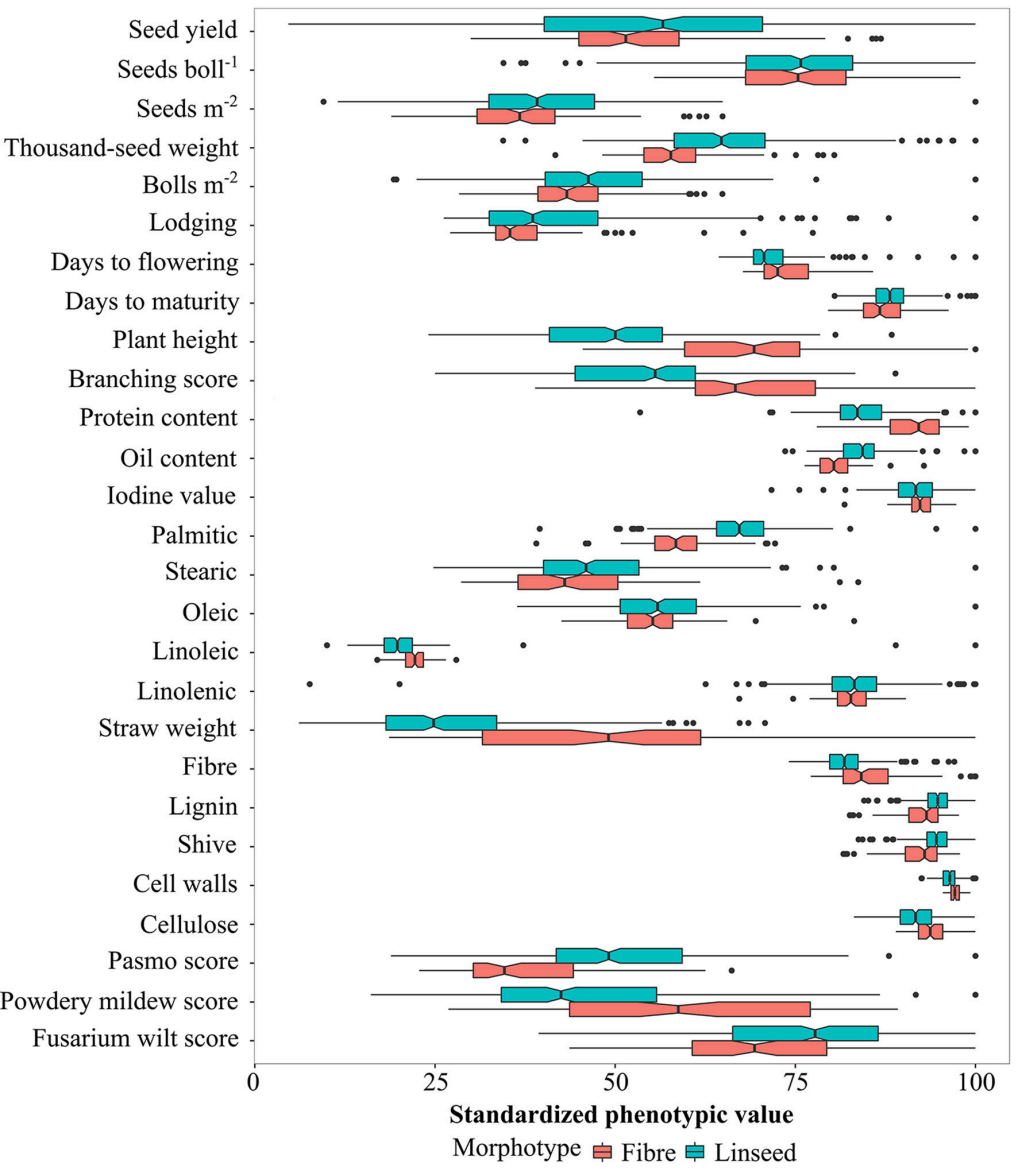
Graphical comparison of variation in linseed and fiber types for 27 traits. The absolute values (see Table S6) have been standardized as percentages of the maximum value of each trait. Statistical significance between linseed and fiber types at the 0.05 probability level is indicated with notched boxes. Box notches that do not overlap indicate median differences between fiber and linseed accessions at a 95% confidence level.

Based on the discriminated morphotypes, a one-way ANOVA was performed to test for significant differences between the fiber (92) and linseed (299) subgroups for the 27 traits. A total of 22 traits, the exceptions being YLD, SEB, STE, OLE, and LIN, showed significant differences between the linseed and fiber flax accessions at the 5% probability level (Figure 3; Table S6). On average, linseed accessions had higher SM2, TSW, BM2, OIL, and they were more resistant to powdery mildew, while fiber accessions had higher STR, PLH, BSC, DTF, and they were more resistant to pasmo and fusarium wilt (Figure 3). However, similarities or overlaps between the two types existed for many traits (Figure 3). Fairly large variations (with $\hat{CV}$ > 15%) were observed within both linseed and fiber groups with respect to YLD, SM2, BM2, LOD, BSC, PLH, STE, STR, and the three disease resistance traits PAS, MIL and WIL (Table S6). SEB, TSW, and OLE also had large variations (>10%) in both groups, and LIO had a large variation (33%) within linseed accessions. Fiber traits, with the exception of STR, had small variations within both morphotypes and within the whole collection.

### Geographical origin of the core collection with phenotypic variation

**Figure 4:**
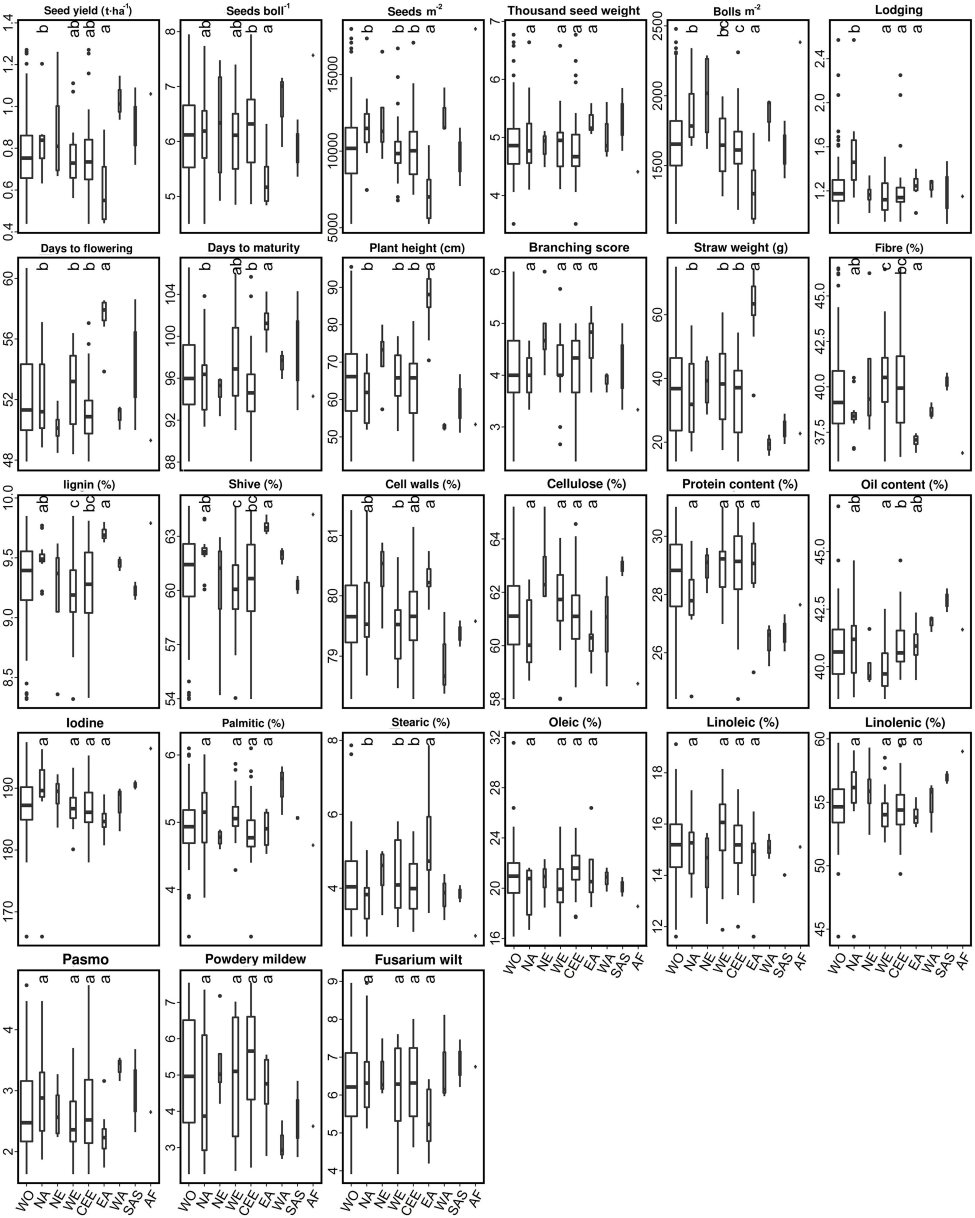
Box plots of 27 traits of the 92 fiber accessions of the core collection in relation to their geographical origin. WO, the whole core collection; NA, North America; SA, South America; NE, Northern Europe; WE, Western Europe; CEE, Central and Eastern Europe; SE, Southern Europe; EA, Eastern Asia; WA, Western Asia; SAS, Southern Asia; OC, Oceania; AF, Africa. Traits are indicated above each graph. Different letters at the top of each boxplot represent statistical significance at the 5% probability level among the four geographical regions with five or more accessions. Box widths are proportional to the sample size of the subsets.

**Figure 5:**
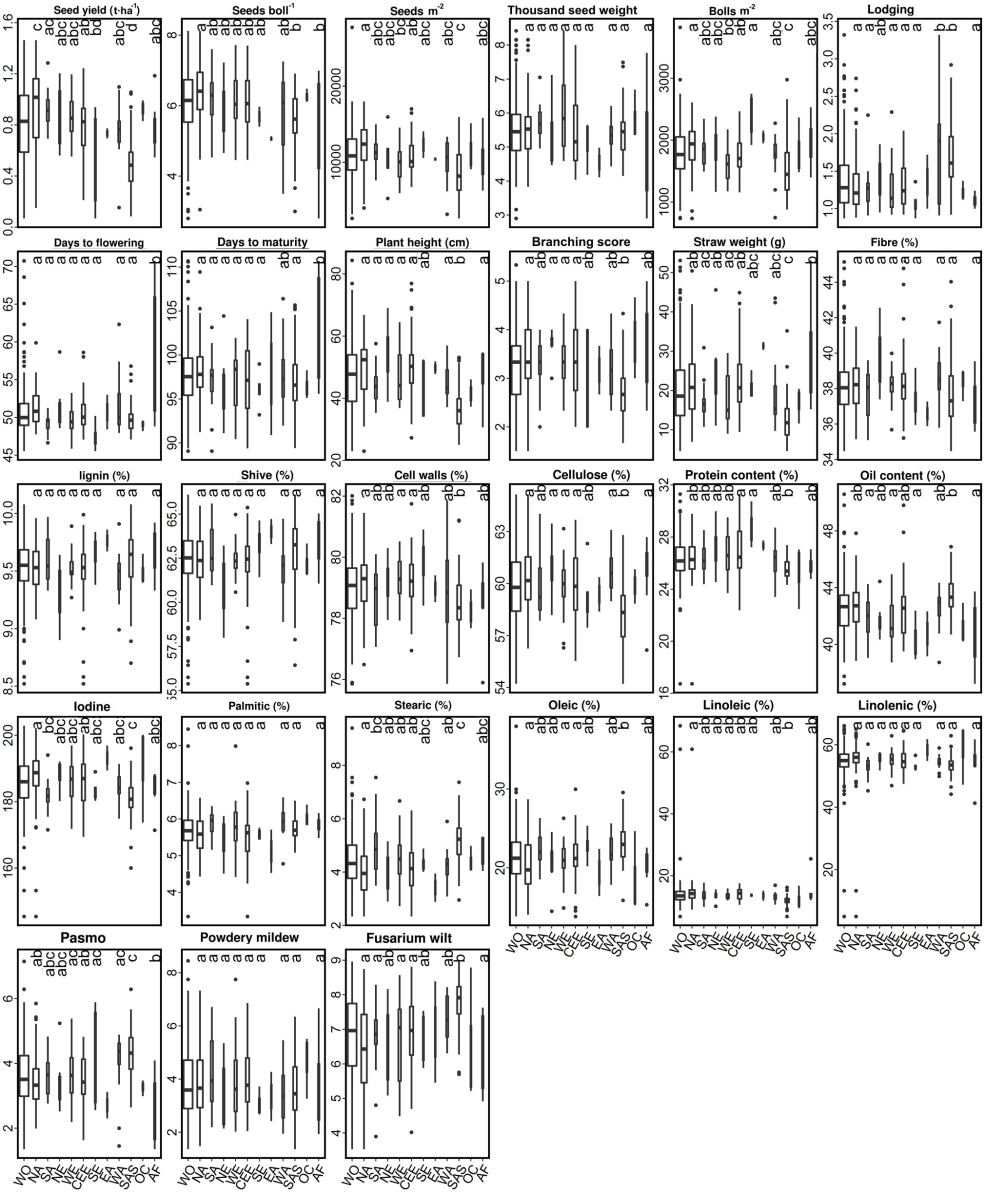
Box plots of 27 traits of the 299 linseed accessions of the core collection in relation to their geographical origin. Different letters at the top of each boxplot represent statistical significance at the 5% probability level among the nine geographical regions with five or more accessions. See Figure 4 for abbreviations and other notes.

**Figure 6:**
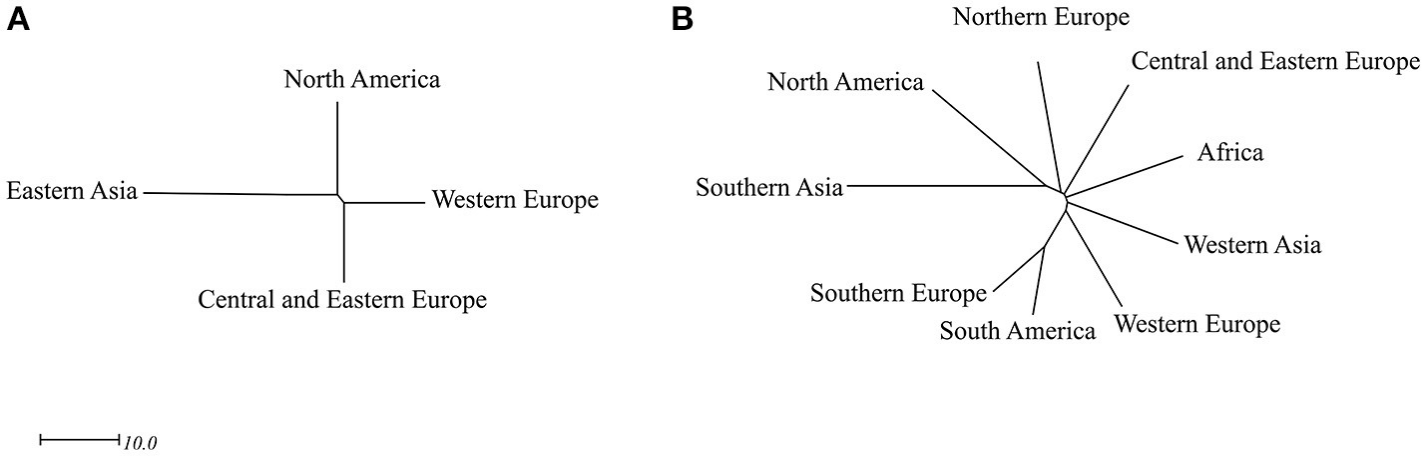
Unrooted trees of the four major geographical origins of the 92 fiber accessions **(A)** and the nine major geographical origins of the 299 linseed accessions **(B)**. The Ward method was used to perform cluster analysis based on the Euclidean distances among the four (Table S7) and nine geographical origins (Table S8) of the two groups, respectively.

The 92 fiber accessions were sampled from eight geographical regions, including CEE (39), WE (22), NA (13), EA (8), NE (4), WA (3), SAS (2), and AF (1), while the 299 linseed accessions were selected from 11 geographical regions, namely NA (119), SAS (52), CEE (46), WE (25), AF (8), NE (7), SE (5), OC (3), and EA (2) (Table S1). The performance of 11 subpopulations of different geographical origins with respect to the 27 traits is depicted by box plots for fiber (Figure 4) and linseed (Figure 5) accessions, respectively. Regions with less than five accessions were excluded from further comparative analyses because of their too small sample sizes. Thus, four and nine geographical regions respectively for fiber and linseed were retained. EA (China and Japan) fiber type accessions differed significantly from those of the other three regions (CEE, WE and NA) for five traits: BM2, DTF, PLH, STR, and STE (Figure 4). The eight fiber accessions from EA had typically higher PLH, DTF, STR, and lower SEB and SM2 than those from the other regions, while no significant differences among the four regions were detected for most traits because of the large within-region variations. Euclidean distances between and within the four geographical regions over the 27 traits further supported these conclusions (Table S7). Accessions from EA and NA (45.03) were the most distinct from one another. NA, WE, and CEE all had high within-region distances (diagonal line in Table S7), close to most pairs of between-region distances, showing large within-region variations. Further cluster analyses based on the distances also demonstrated the large difference between EA and the other three regions (NA, WE, and CEE) (Figure 6A).

For linseed accessions, significant differences between at least two of the nine geographical regions were observed in 20 of the 27 traits, the exception being TSW, FIB, LIG, SHI, PAL, LIN, and MIL (Figure 5). However, these differences existed primarily between SAS and the other regions. On average, the 52 accessions from SAS had significantly lower YLD, PLH, BSC, STR, and higher LOD scores (even short plants) (Figure 5). Accessions from NA had relatively high YLD, SEB, SM2 and low LOD. Accessions from AF were late flowering and maturing while those from SE were early flowering. However, all regions had high within-region variations (diagonal line in Table S8), and the within-region distances were even larger than some pairs of between-region distances (Table S8). The average Euclidean distance of 30.15 ± 8.03 within geographical regions (diagonal line in Table S8) was similar to the average distance between geographical regions of 31.52 ± 5.39. The greatest diversity existed within NE (38.25), followed by SAS (36.21), AF (35.27), NA (33.87), WA (33.39), and CEE (33.22) which had similar diversity. Accessions from NA and SA were relatively more distinct from those of the other regions, averaging 37.67 ± 3.57 and 35.01 ± 5.39, respectively. The largest distinction (44.76) was observed between SAS and NA (Table S8). Further cluster analyses based on the distances also showed that SAS was distinct from the other regions (Figure 6B).

### Cluster analysis

**Figure 7:**
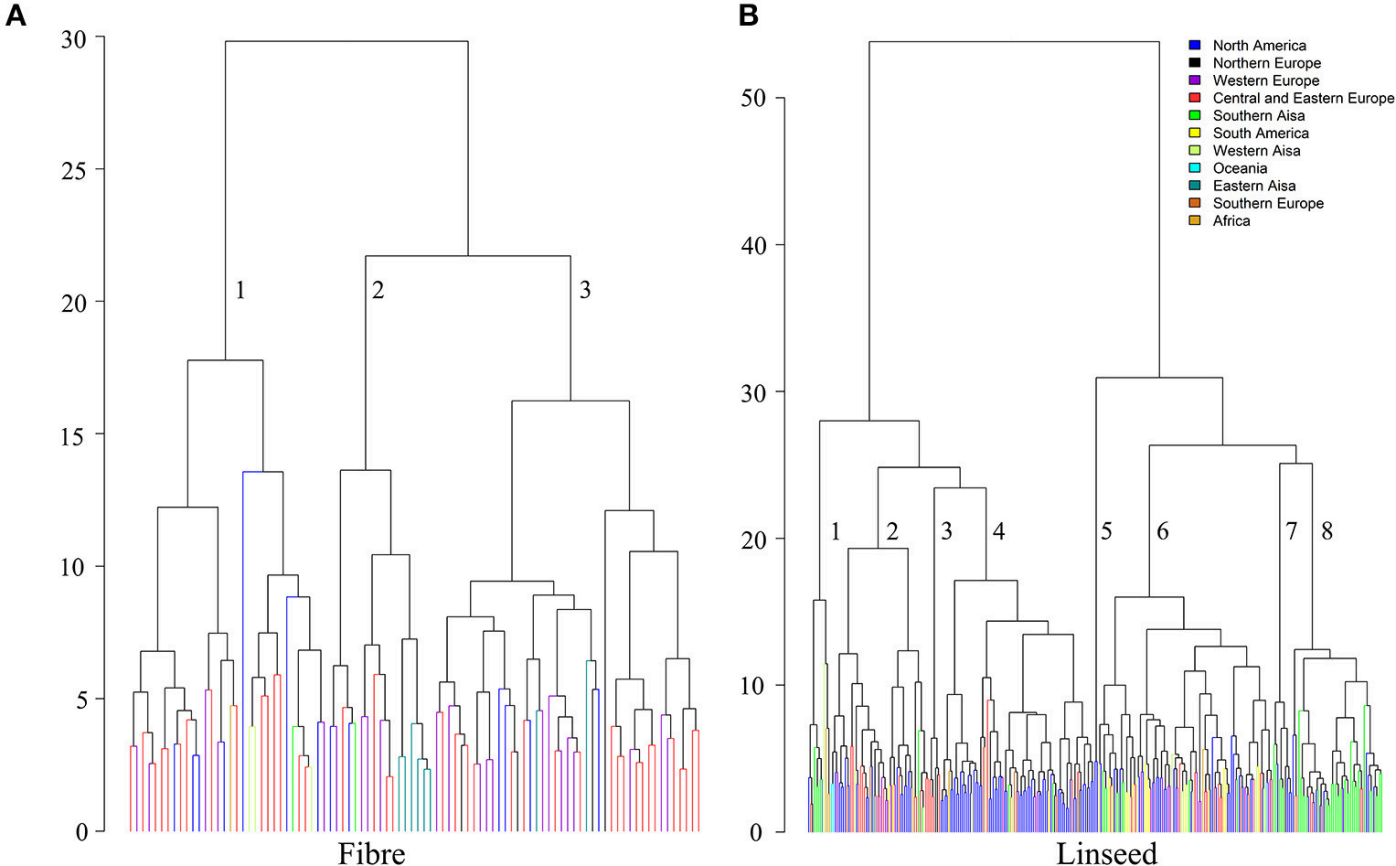
Dendrograms derived from cluster analyses of the 92 fiber **(A)** and 299 linseed accessions **(B)** of the flax core collection. The Ward method was used for cluster analysis. Accessions are colored based on geographical origin.

Hierarchical cluster analysis was performed separately for fiber and linseed accessions. According to the means and distances between and within clusters, the 92 fiber accessions grouped into three clusters (Figure 7A; Table S9). Characteristics of the three clusters containing 32, 17, and 43 accessions, respectively, are summarized in Table 2. Cluster 1 contained accessions with characteristics similar to linseed accessions with relatively high yield and short stature (Table 2 and Table S9). These accessions are important resources for breeding of intermediate type and dual purpose flax. Cluster 2 comprised all highly typical fiber accessions with high straw weight, plant height and low yield. This cluster contained six of the eight cultivars from EA. These accessions are best suited for fiber variety improvement. The accessions in cluster 3 had intermediate characteristics between clusters 1 and 2. All three clusters contained accessions originating from different geographical regions, indicative of a weak relationship between trait performance and geographical origins.

**Table 2:** Summary of cluster analysis for 92 fiber accessions with 27 traits.

**Cluster**	**No. of accessions**	**Geographical origin**	**Major characteristics**
1	32	Europe (20), North America (7), Asia (4) and Africa (1)	Low straw weight and branching score, short plants, early flowering; relatively high seed yield, seeds m^−2^ and bolls m^−2^. Somewhat similar to linseed accessions.
2	17	Europe (8), Asia (7) and North America (2)	Typical fiber accessions. High straw weight, plant height and branching score; late flowering and maturity. Very low seed yield, seeds m^−2^ and bolls m^−2^ but large seeds.
3	43	Europe (37), North America (4) and Asia (2)	Intermediate trait performance between clusters 1 and 2. Moderate straw weight but high fiber and low lignin and shive contents. Susceptible to powdery mildew but resistant to fusarium wilt.

*Major characteristics of the three clusters are summarized based on trait comparison in Table S9*.

The 299 linseed accessions grouped into eight clusters (Figure 7B; Table S10). The clusters' composition including geographical origins and major characteristics of the accessions are summarized in Table 3. Clusters 4 and 5 contained all high-yielding modern flax cultivars or breeding lines that are primarily from NA, such as CDC Bethune and Macbeth. Cluster 5 contained only two modern cultivars: Linola 989 and CDC Gold. These two Canadian cultivars are special seed quality types with low LIN (9.1%) but high LIO (64.7%) (You et al., 2016b) (Table S10). The accessions from these two clusters constitute elite adapted germplasm for linseed, particularly for NA. Cluster 7 contained ten accessions from SAS (5), NA (4), and NE (1) representing a germplasm with very large seeds (TSW of 7.13 ± 0.55 g), early flowering (DTF of 48.33 ± 0.66 days) and maturing (DTM of 93.34 ± 2.84 days), and high oil content (43.80 ± 2.11%). Cluster 8 (47 accessions primarily from SAS) comprises accessions that are also early flowering (49.00 ± 1.24 days) and maturing (96.35 ± 2.49 days) and high oil content (43.32 ± 1.20 %). Both clusters 7 and 8's accessions were quite low yielding, susceptible to fusarium wilt and short (32.37 ± 6.22 cm and 35.08 ± 4.15 cm for Cluster 7 and 8, respectively) and thus represent a source for short plant height genes. The six accessions of cluster 3 had characteristics similar to fiber type, i.e., accessions were tall and had high straw weight and fiber content, while the 12 accessions of cluster 1 were characterized by high straw weight (32.42 ± 10.09 g), the latest flowering and maturity times and the highest LIN (59.73 ± 4.28 %) compared to the other clusters, but were of average height. Accessions from these two clusters constitute useful germplasm for dual purpose flax breeding. Cluster 2 includes another set of germplasm for early flowering and maturity as well as high LIN. Cluster 6, with 91 accessions, is the largest cluster of linseed cultivars and lines originating primarily from Europe, North America and Asia. These accessions had moderate seed yield but larger seeds (6.05 ± 0.94 g) than accessions in any other clusters except for cluster 7. All cluster information for fiber and linseed types obtained from the cluster analyses is listed in Table S2.

**Table 3:** Summary of cluster analysis for 299 linseed accessions with 27 traits.

**Cluster**	**No. of accessions**	**Geographical origin**	**Major characteristics**
1	12	Asia (6), Africa (3), Europe (2) and North America (1)	Low seed yield with high bolls m^−2^, low seeds boll^−1^ and small seeds; late flowering and maturity; high linolenic acid content; high straw weight; susceptible to fusarium wilt.
2	51	Europe (24), North America (17), Asia (5), and others (5)	Moderate seed yield with small seeds but high number of seeds boll^−1^; early flowering and maturity; high linolenic acid content.
3	6	Central and Eastern Europe (4) and Northern Europe (2)	Low seed yield; tall, high straw weight and fiber content. Moderately resistant to pasmo. Somewhat similar to fiber accessions.
4	80	North America (59), Europe (13), Asia (4), South America (2) and Africa (2)	High-yielding modern cultivars and breeding lines. High seed yield, seeds m^−2^ and bolls m^−2^; moderate flowering and maturity, seed size and plant height; resistant to lodging; moderately resistant to pasmo and powdery mildew.
5	2	North America (2)	High linoleic acid content and seed yield; modern Canadian cultivars.
6	91	North America (26), Europe (44), Asia (18), Africa (2) and Oceania (1)	Moderate seed yield with large seeds but low seeds m^−2^; high oleic acid content; relatively low straw weight; susceptible to fusarium wilt.
7	10	Southern Asia (5), North America (4) and Northern Europe (1)	Very low seed yield, seeds m^−2^ and bolls m^−2^ but very large seed size; very short plants and low straw weight; early flowering and maturity; high oil and oleic acid contents; susceptible to fusarium wilt.
8	47	Southern Asia (30), North America (10) and Europe (7)	Very low seed yield with moderate seed size; very short plants and low straw weight; early flowering and maturity; very high oleic acid content and high oil; susceptible to fusarium wilt.

*Major characteristics of the eight clusters are summarized based on trait comparison in Table S10*.

## Discussion

### Genetic variability of the core collection and breeding applications

A core collection consists of a limited number of accessions that represent the breadth of the genetic diversity of a large whole germplasm collection of a given crop (van Hintum et al., 2000). For more than 35 years, the PGRC has obtained and evaluated flax accessions from many countries (Diederichsen, 2007; Diederichsen and Fu, 2008). A core collection of 381 flax accessions augmented with an additional 26 modern breeding lines and cultivars was recently assembled (Diederichsen et al., 2013; Soto-Cerda et al., 2013) based on phenotypic data of the accessions, rather than random selection, to maximize the diversity and preserve the range of variation in the whole collection (Diederichsen et al., 2013). The genetic diversity of this core collection has been assessed at the molecular level using microsatellite or simple sequence repeat (SSR) markers, revealing an abundant genetic diversity among the accessions with an average of 5.32 alleles per locus over 414 SSRs (Soto-Cerda et al., 2013).

In the present study, ten agronomic, eight seed quality, six fiber and three disease resistance traits of importance to both breeders and growers were assessed in up to eight environments (years and locations). This study represents the most comprehensive assessment of phenotypic performance of this flax core collection to date. The observations in multiple environments will be useful for breeding selection, genetic diversity evaluation, association mapping studies and genomic selection. The study revealed the large genetic variability and the selection potential for most traits, especially seed yield, straw weight (an indicator of fiber yield), disease resistance and other agronomic traits through $\hat{CV}$ or $\hat{GCV}$ and Δ*G*. Compared to the previously reported data (values in parentheses in Table 1; Diederichsen et al., 2013), the core collection was estimated to have a slightly lower variation for TSW, DTF, PLH, and FAC (PAL, STE and OLE). With the exception of fiber content (low $\hat{CV}\;$of 5.2% compared to 16.0% for the whole PGRC collection), variations in OIL, LIN, and LIO were significantly increased by the addition of a few modern breeding lines and cultivars to the core collection. Thus, the core collection represents the majority of the variation of the whole collection and provides diverse germplasm for flax breeding. The cluster analyses grouped the 92 fiber accessions into three clusters and the 299 linseed accessions into eight clusters. The accessions in each cluster have defining characteristics such as high yield, early flowering and maturity, high stature and biomass, high linolenic acid content, large seeds and disease resistance, that defines them as resources for specific breeding purposes. Despite the large variability for disease traits, only few accessions were highly resistant to any of the diseases (Table 1). Consequently, additional resistant germplasm is still required to enhance this core collection for breeding and genetic studies of resistance to pasmo, fusarium wilt and powdery mildew.

### Divergence between fiber flax and linseed

Phylogenetic analyses supported the hypothesis of a single domestication origin of pale flax as the wild progenitor, first domesticated for its oil rather than fiber use (Allaby et al., 2005; Fu and Allaby, 2010). New archaeological evidence based on archaeobotanical datasets of flax seed sizes in the Late Neolithic also suggests that flax for fiber was cultivated at a later date (Herbig and Maier, 2011). The divergence between linseed and fiber flax is the result of long term disruptive selection for the different end uses of the crop (Soto-Cerda et al., 2013). Long term artificial selection for fiber or linseed flax by Neolithic farmers would have been based on morphological and agronomic traits, such as plant height, branching architecture, flowering and maturity times, biomass, seed yield, and yield components because the differences between the two types of flax lie primarily in morphological and agronomic traits rather than fatty acid composition (Figure 3; Table S6).

Despite the divergence between fiber and linseed types, we noticed that only 17 out of the 92 fiber accessions (Cluster 2 in Table 2) were highly typical of fiber cultivars while only 82 out of the 299 linseed accessions (Clusters 4 and 5 in Table 3) could be considered to have typical modern linseed cultivar attributes. Many fiber and linseed accessions shared similar trait performance (Figure 3) and had characteristics of both fiber and linseed types. For example, some fiber accessions had seed yield similar to linseed accessions (Cluster 1 in fiber accessions, Table 2), and vice-versa (Cluster 1 and 3 for linseed accessions, Table 3). These accessions may be of an intermediate type, constituting useful parents for the development of dual purpose cultivars.

### Geographical patterns of variability of the core collection

The 391 accessions of the core collection from 38 countries were grouped into 11 geographical regions (Tables S1 and S2). Separate analyses were performed for two morphotypes because of the divergence between fiber and linseed accessions. PCA and Euclidean distances between geographical regions demonstrated weak geographical patterns in the core collection with the exception of East Asia for fiber type and Southern Asia and North America for linseed type. The fiber accessions that originated from East Asia were tall, with few branches, high straw weight and low yield which are typical characteristics of the fiber type but different from the fiber accessions of the other regions. The majority of the linseed accessions from North America were high-yielding modern cultivars while most of the linseed accessions from Southern Asia were low-yielding with short stature. These were also significantly different from the accessions from other regions.

Several studies were performed at the molecular level. Fu (2005) used 67 random amplified polymorphic DNA (RAPD) markers producing 149 scored RAPD bands to assess 2,727 flax accessions of the PGRC collection, which comprised most of the accessions of the core collection. Only 8.2% of the RAPD variation was explained by the geographical origin, an estimate similar to the 11.0% we obtained from our phenotypic evaluations. Based on genetic structure analysis with 448 SSR markers, Soto-Cerda et al. (2013) assigned all 407 accessions in the core collection to two major groups and six sub-groups. Weak population differentiation was observed between major groups and most sub-groups, indicating a weak population structure that is suitable for association mapping studies (Soto-Cerda et al., 2013, 2014).

## Conclusion

We assessed the genetic variability of 27 traits of a flax core collection evaluated in up to 8 year-location environments. Large variability for most traits was quantified in both fiber and linseed accessions. Both divergence and similarity between fiber and oil morphotypes should help breeder's decision toward the development of fiber, linseed or dual purpose varieties. Weak patterns among geographical regions were observed but, more importantly, germplasm with specific characteristics was identified and clustered. This data will guide breeders toward better educated decision of germplasm utilization in flax genetic improvement. The phenotypic evaluation of 27 traits over multiple environments constitutes a valuable resource for breeding selection, genetic diversity evaluation, association studies and genomic selection.

## Author contributions

SC, SD, KR, HB, and FY conceived and designed the study. SD, KR, and HB implemented field trials and performed the phenotyping. FY, GJ, and JX performed data analysis and prepared tables and figures. FY and JX drafted the manuscript. All authors reviewed and edited the manuscript.

### Conflict of interest statement

The authors declare that the research was conducted in the absence of any commercial or financial relationships that could be construed as a potential conflict of interest.
