# Supplementary material for: Genetic Variability of 27 Traits in a Core Collection of Flax (Linum usitatissimum L.)
Source: Front Plant Sci. 2017 Sep 21;8:1636. doi: 10.3389/fpls.2017.01636 (PMC5622609; doi:10.3389/fpls.2017.01636)
Supplement: Supplementary file 1 [file Table1.docx]

**TABLE S1** Summary of germplasm in the flax core collection by geographical origin, improvement status and morphotype.

| **Region** | **Country** |  |  | | **Number of accessions** | | | | | | | | | |
| --- | --- | --- | --- | --- | --- | --- | --- | --- | --- | --- | --- | --- | --- | --- |
|  |  | **Improvement status** | | | | | |  | **Morphotype** | | | | | **Total** |
|  |  | **Cultivar** | | **Breeding line** | | **Landrace** | **Un^.#^** |  | **Fibre** | **Linseed** | **Un. ^#^** | **Fibre†** | **Linseed†** |  |
| North America | Canada | 36 | | 31 | | - | - | 3 | | 61 | 3 | 4 | 63 | 67 |
|  | United States | 45 | | 20 | | - | - | 8 | | 47 | 10 | 9 | 56 | 65 |
|  | **Subtotal** | **81** | | **51** | | **-** | **-** | **11** | | **108** | **13** | **13** | **119** | **132** |
| South America | Argentina | 15 | | 2 | | - | - | - | | 16 | 1 | - | 17 | 17 |
|  | Uruguay | 1 | | - | | - | - | - | | 1 | - | - | 1 | 1 |
|  | **Subtotal** | **16** | | **2** | | **-** | **-** | **-** | | **17** | **1** | **-** | **18** | **18** |
| Eastern Asia | China | 1 | | 7 | | - | - | 7 | | - | 1 | 7 | 1 | 8 |
|  | Japan | 2 | | - | | - | - | 1 | | 1 | - | 1 | 1 | 2 |
|  | **Subtotal** | **3** | | **7** | | **-** | **-** | **8** | | **1** | **1** | **8** | **2** | **10** |
| Southern Asia | Afghanistan | 1 | | 1 | | - | 2 | 1 | | 3 | - | 1 | 3 | 4 |
|  | India | 26 | | - | | 4 | 3 | - | | 32 | 1 | - | 33 | 33 |
|  | Iran | 4 | | - | | 2 | - | 1 | | 5 | - | 1 | 5 | 6 |
|  | Pakistan | 9 | | - | | 2 | - | - | | 10 | 1 | - | 11 | 11 |
|  | **Sub total** | **40** | | **1** | | **8** | **5** | **2** | | **50** | **2** | **2** | **52** | **54** |
| Western Asia | Armenia | - | | - | | - | 1 | - | | 1 | - | - | 1 | 1 |
|  | Georgia | - | | - | | - | 2 | - | | 2 | - | - | 2 | 2 |
|  | Cyprus | - | | - | | - | 1 | - | | 1 | - | - | 1 | 1 |
|  | Turkey | 5 | | - | | 1 | 7 | 3 | | 9 | 1 | 3 | 10 | 13 |
|  | **Subtotal** | **5** | | **-** | | **1** | **11** | **3** | | **13** | **1** | **3** | **14** | **17** |
| Oceania | Australia | 1 | | - | | - | - | - | | 1 |  | - | 1 | 1 |
|  | New Zealand | - | | 2 | | - | - | - | |  | 2 | - | 2 | 2 |
|  | **Sub total** | **1** | | **2** | | **-** | **-** | **-** | | **1** | **2** | **-** | **3** | **3** |
| Western Europe | France | 17 | | 3 | | 2 | - | 7 | | 14 | 1 | 7 | 15 | 22 |
|  | Germany | 3 | | 2 | | - | - | - | | 5 | - | - | 5 | 5 |
|  | Netherlands | 17 | | - | | 2 | - | 14 | | 5 | - | 14 | 5 | 19 |
|  | Estonia | - | | - | | - | 1 | - | | - | 1 | 1 | 0 | 1 |
|  | **Subtotal** | **37** | | **5** | | **4** | **1** | **21** | | **24** | **2** | **22** | **25** | **47** |
| Central and Eastern Europe | Belarus | 1 | | - | | - | - | 1 | | - | - | 1 | - | 1 |
|  | Czech Republic | 6 | | - | | - | - | 1 | | 4 | 1 | 1 | 5 | 6 |
|  | Czechoslovakia | 1 | | - | | - | - | - | | 1 | - | - | 1 | 1 |
|  | Hungary | 13 | | - | | - | - | 3 | | 10 | - | 3 | 10 | 13 |
|  | Poland | 1 | | 1 | | - | - | 1 | | 1 | - | 1 | 1 | 2 |
|  | Romania | 3 | | - | | - | 3 | 3 | | 2 | 1 | 3 | 3 | 6 |
|  | Russian | 18 | | 17 | | 6 | 9 | 26 | | 23 | 1 | 26 | 24 | 50 |
|  | Ukraine | 2 | | - | | - | 3 | 4 | | 1 | - | 4 | 1 | 5 |
|  | Slovenia | 1 | | - | | - | - | - | | 1 | - | - | 1 | 1 |
|  | **Subtotal** | **46** | | **18** | | **6** | **15** | **39** | | **43** | **3** | **39** | **46** | **85** |
| Southern Europe | Portugal | 2 | | - | | 1 | - | - | | 1 | 2 | - | 3 | 3 |
|  | Greece | - | | - | | - | 2 | - | | 1 | 1 | 0 | 2 | 2 |
|  | **Subtotal** | **2** | | **-** | | **1** | **2** | **-** | | **2** | **3** | **0** | **5** | **5** |
| Northern Europe | Ireland | 1 | | - | | - | - | - | | 1 | - | - | 1 | 1 |
|  | United Kingdom | 5 | | - | | - | 1 | 2 | | 4 | - | 2 | 4 | 6 |
|  | Sweden | 1 | | - | | - | - | 1 | | - | - | 1 | - | 1 |
|  | Lithuania | 1 | | 2 | | - | - | 1 | | 2 | - | 1 | 2 | 3 |
|  | **Subtotal** | **8** | | **2** | | **-** | **1** | **4** | | **7** | **-** | **4** | **7** | **11** |
| Africa | Egypt | 1 | | - | | - | 1 | 1 | | 1 | - | 1 | 1 | 2 |
|  | Morocco | 1 | | 1 | | - | - | - | | 1 | 1 | - | 2 | 2 |
|  | Ethiopia | 4 | | 1 | | - | - | - | | 5 | - | - | 5 | 5 |
|  | **Subtotal** | **6** | | **2** | | **-** | **1** | **1** | | **7** | **1** | **1** | **8** | **9** |
|  | **Total** | **245** | | **90** | | **20** | **36** | **89** | | **273** | **29** | **92** | **299** | **391** |

^#^ Un., Unknown morphotype. †The number of fibre and linseed accessions after discrimination assignment of morphotype.
