# Supplementary material for: Genetic Variability of 27 Traits in a Core Collection of Flax (Linum usitatissimum L.)
Source: Front Plant Sci. 2017 Sep 21;8:1636. doi: 10.3389/fpls.2017.01636 (PMC5622609; doi:10.3389/fpls.2017.01636)
Supplement: Supplementary file 2 [file Table2.DOCX]

**TABLE S2** List of the germplasm of the flax core collection and clusters obtained from the cluster analysis.

| **Accession** | **ID** | | **Morphotype** | **Predicted morphotype** | **Improvement status** | **Country** | **Country abbrev.** | **Geographical region** | **Region abbrev.** | **Order in dendrogram** | **Cluster** |
| --- | --- | --- | --- | --- | --- | --- | --- | --- | --- | --- | --- |
| CN97531 | | TMP-2161 | Fibre | Fibre | Landrace | Russian Federation | RUS | Central and Eastern Europe | CEE | 1 | 1 |
| CN97610 | | TMP-2170 | Fibre | Fibre | Cultivar | Netherlands | NLD | Western Europe | WE | 2 | 1 |
| CN101154 | | TMP-9786 | Fibre | Fibre | Cultivar | Russian Federation | RUS | Central and Eastern Europe | CEE | 3 | 1 |
| CN100795 | | TMP-10250 | Fibre | Fibre | Cultivar | Netherlands | NLD | Western Europe | WE | 4 | 1 |
| CN97530 | | TMP-2160 | Fibre | Fibre | Landrace | Russian Federation | RUS | Central and Eastern Europe | CEE | 5 | 1 |
| CN101379 | | TMP-13179 | Fibre | Fibre | Unknown | Ukraine | UKR | Central and Eastern Europe | CEE | 6 | 1 |
| CN101397 | | TMP-13197 | Fibre | Fibre | Unknown | Ukraine | UKR | Central and Eastern Europe | CEE | 7 | 1 |
| CN98934 | | TMP-2230 | Fibre | Fibre | Cultivar | United States | USA | North America | NA | 8 | 1 |
| CN98286 | | TMP-2269 | Fibre | Fibre | Cultivar | Hungary | HUN | Central and Eastern Europe | CEE | 9 | 1 |
| CN101364 | | TMP-13163 | Fibre | Fibre | Unknown | Russian Federation | RUS | Central and Eastern Europe | CEE | 10 | 1 |
| CN98829 | | TMP-2156 | Fibre | Fibre | Cultivar | United States | USA | North America | NA | 11 | 1 |
| CN97452 | | TMP-7529 | Unknown | Fibre | Cultivar | United States | USA | North America | NA | 12 | 1 |
| CN97424 | | TMP-2147 | Fibre | Fibre | Cultivar | Netherlands | NLD | Western Europe | WE | 13 | 1 |
| CN97503 | | TMP-7573-6 | Fibre | Fibre | Landrace | Russian Federation | RUS | Central and Eastern Europe | CEE | 14 | 1 |
| CN97616 | | TMP-7670 | Fibre | Fibre | Cultivar | Netherlands | NLD | Western Europe | WE | 15 | 1 |
| CN97665 | | TMP-7702-5 | Fibre | Fibre | Breeding line | United States | USA | North America | NA | 16 | 1 |
| CN98826 | | TMP-7516 | Fibre | Fibre | Cultivar | Egypt | EGY | Africa | AF | 17 | 1 |
| CN97533 | | TMP-7619-2 | Fibre | Fibre | Landrace | Russian Federation | RUS | Central and Eastern Europe | CEE | 18 | 1 |
| CN98923 | | TMP-2222-4 | Fibre | Fibre | Breeding line | United States | USA | North America | NA | 19 | 1 |
| CN101382 | | TMP-13182 | Fibre | Fibre | Unknown | Turkey | TUR | Western Asia | WA | 20 | 1 |
| CN101386 | | TMP-13186 | Fibre | Fibre | Unknown | Turkey | TUR | Western Asia | WA | 21 | 1 |
| CN32542 | | PGR-4044 | Fibre | Fibre | Cultivar | Russian Federation | RUS | Central and Eastern Europe | CEE | 22 | 1 |
| CN98303 | | TMP-2275 | Fibre | Fibre | Cultivar | Hungary | HUN | Central and Eastern Europe | CEE | 23 | 1 |
| CN101402 | | TMP-13203 | Fibre | Fibre | Unknown | Russian Federation | RUS | Central and Eastern Europe | CEE | 24 | 1 |
| CN98704 | | TMP-8230 | Fibre | Fibre | Cultivar | Czech Republic | CZE | Central and Eastern Europe | CEE | 25 | 1 |
| CN101486 | | TMP-9935 | Fibre | Fibre | Breeding line | Canada | CAN | North America | NA | 26 | 1 |
| CN100952 | | TMP-1654 | Fibre | Fibre | Unknown | Afghanistan | AFG | Southern Asia | SAS | 27 | 1 |
| CN101378 | | TMP-13178 | Fibre | Fibre | Unknown | Ukraine | UKR | Central and Eastern Europe | CEE | 28 | 1 |
| CN101348 | | TMP-13147 | Fibre | Fibre | Unknown | Russian Federation | RUS | Central and Eastern Europe | CEE | 29 | 1 |
| CN101385 | | TMP-13185 | Fibre | Fibre | Unknown | Turkey | TUR | Western Asia | WA | 30 | 1 |
| CN100848 | | TMP-1419 | Fibre | Fibre | Cultivar | Canada | CAN | North America | NA | 31 | 1 |
| CN98710 | | TMP-8238-10 | Fibre | Fibre | Landrace | France | FRA | Western Europe | WE | 32 | 1 |
| CN101572 | | TMP-10118 | Unknown | Fibre | Breeding line | Canada | CAN | North America | NA | 33 | 2 |
| CN98708 | | TMP-8233 | Fibre | Fibre | Cultivar | France | FRA | Western Europe | WE | 34 | 2 |
| CN100864 | | TMP-1437 | Fibre | Fibre | Cultivar | Hungary | HUN | Central and Eastern Europe | CEE | 35 | 2 |
| CN101559 | | TMP-10091 | Fibre | Fibre | Breeding line | Canada | CAN | North America | NA | 36 | 2 |
| CN97180 | | TMP-2289 | Fibre | Fibre | Cultivar | Iran | IRN | Southern Asia | SAS | 37 | 2 |
| CN101327 | | TMP-13126 | Unknown | Fibre | Unknown | Estonia | EST | Western Europe | WE | 38 | 2 |
| CN97351 | | TMP-8236 | Fibre | Fibre | Cultivar | France | FRA | Western Europe | WE | 39 | 2 |
| CN18991 | | TMP-1160 | Fibre | Fibre | Cultivar | Poland | POL | Central and Eastern Europe | CEE | 40 | 2 |
| CN18983 | | TMP-1152 | Fibre | Fibre | Cultivar | Netherlands | NLD | Western Europe | WE | 41 | 2 |
| CN101403 | | TMP-13210 | Fibre | Fibre | Unknown | Romania | ROM | Central and Eastern Europe | CEE | 42 | 2 |
| CN101404 | | TMP-13211 | Fibre | Fibre | Unknown | Romania | ROM | Central and Eastern Europe | CEE | 43 | 2 |
| CN101416 | | TMP-1919 | Fibre | Fibre | Breeding line | China | CHN | Eastern Asia | EA | 44 | 2 |
| CN101419 | | TMP-1922 | Fibre | Fibre | Breeding line | China | CHN | Eastern Asia | EA | 45 | 2 |
| CN101052 | | TMP-1791 | Fibre | Fibre | Breeding line | China | CHN | Eastern Asia | EA | 46 | 2 |
| CN101053 | | TMP-1792 | Fibre | Fibre | Breeding line | China | CHN | Eastern Asia | EA | 47 | 2 |
| CN101230 | | TMP-10803 | Fibre | Fibre | Breeding line | China | CHN | Eastern Asia | EA | 48 | 2 |
| CN101421 | | TMP-1924 | Fibre | Fibre | Breeding line | China | CHN | Eastern Asia | EA | 49 | 2 |
| CN18987 | | TMP-1156 | Fibre | Fibre | Cultivar | Netherlands | NLD | Western Europe | WE | 50 | 3 |
| CN101055 | | TMP-1794 | Fibre | Fibre | Breeding line | Russian Federation | RUS | Central and Eastern Europe | CEE | 51 | 3 |
| CN101392 | | TMP-13192 | Fibre | Fibre | Cultivar | France | FRA | Western Europe | WE | 52 | 3 |
| CN101401 | | TMP-13202 | Fibre | Fibre | Unknown | Russian Federation | RUS | Central and Eastern Europe | CEE | 53 | 3 |
| CN101118 | | TMP-1857 | Fibre | Fibre | Breeding line | Lithuania | LTU | Northern Europe | NE | 54 | 3 |
| CN101119 | | TMP-1858 | Fibre | Fibre | Breeding line | Russian Federation | RUS | Central and Eastern Europe | CEE | 55 | 3 |
| CN101406 | | TMP-13213 | Fibre | Fibre | Unknown | Russian Federation | RUS | Central and Eastern Europe | CEE | 56 | 3 |
| CN101407 | | TMP-13214 | Fibre | Fibre | Unknown | Netherlands | NLD | Western Europe | WE | 57 | 3 |
| CN40081 | | PGR-13075 | Fibre | Fibre | Cultivar | Netherlands | NLD | Western Europe | WE | 58 | 3 |
| CN33390 | | PGR-5041 | Fibre | Fibre | Cultivar | Netherlands | NLD | Western Europe | WE | 59 | 3 |
| CN98903 | | TMP-2202-8 | Fibre | Fibre | Breeding line | United States | USA | North America | NA | 60 | 3 |
| CN98954 | | TMP-2261 | Fibre | Fibre | Cultivar | United States | USA | North America | NA | 61 | 3 |
| CN32546 | | PGR-4048 | Fibre | Fibre | Cultivar | Ukraine | UKR | Central and Eastern Europe | CEE | 62 | 3 |
| CN33393 | | PGR-5044 | Fibre | Fibre | Cultivar | United Kingdom | UNK | Northern Europe | NE | 63 | 3 |
| CN101116 | | TMP-1855 | Fibre | Fibre | Breeding line | Russian Federation | RUS | Central and Eastern Europe | CEE | 64 | 3 |
| CN98926 | | TMP-2224-10 | Fibre | Fibre | Breeding line | United States | USA | North America | NA | 65 | 3 |
| CN98072 | | TMP-2187 | Fibre | Fibre | Cultivar | Japan | JPN | Eastern Asia | EA | 66 | 3 |
| CN98150 | | TMP-2192 | Fibre | Fibre | Unknown | Netherlands | NLD | Western Europe | WE | 67 | 3 |
| CN18988 | | TMP-1157 | Fibre | Fibre | Cultivar | France | FRA | Western Europe | WE | 68 | 3 |
| CN101405 | | TMP-13212 | Fibre | Fibre | Unknown | Romania | ROM | Central and Eastern Europe | CEE | 69 | 3 |
| CN100929 | | TMP-1614 | Fibre | Fibre | Cultivar | Netherlands | NLD | Western Europe | WE | 70 | 3 |
| CN18998 | | TMP-1168 | Fibre | Fibre | Cultivar | Netherlands | NLD | Western Europe | WE | 71 | 3 |
| CN19001 | | TMP-1171 | Fibre | Fibre | Cultivar | Netherlands | NLD | Western Europe | WE | 72 | 3 |
| CN101136 | | TMP-1876 | Fibre | Fibre | Cultivar | Russian Federation | RUS | Central and Eastern Europe | CEE | 73 | 3 |
| CN101417 | | TMP-1920 | Fibre | Fibre | Breeding line | China | CHN | Eastern Asia | EA | 74 | 3 |
| CN97871 | | TMP-2177-7 | Fibre | Fibre | Cultivar | Sweden | SWE | Northern Europe | NE | 75 | 3 |
| CN98946 | | TMP-2242 | Fibre | Fibre | Cultivar | United States | USA | North America | NA | 76 | 3 |
| J.W.S. | | J.W.S. | Fibre | Fibre | Cultivar | United Kingdom | UNK | Northern Europe | NE | 77 | 3 |
| CN101038 | | TMP-1777 | Fibre | Fibre | Cultivar | Belarus | BLR | Central and Eastern Europe | CEE | 78 | 3 |
| CN35791 | | PGR-8233 | Fibre | Fibre | Cultivar | Russian Federation | RUS | Central and Eastern Europe | CEE | 79 | 3 |
| CN101127 | | TMP-1866 | Fibre | Fibre | Breeding line | Russian Federation | RUS | Central and Eastern Europe | CEE | 80 | 3 |
| CN18997 | | TMP-1167 | Fibre | Fibre | Cultivar | Netherlands | NLD | Western Europe | WE | 81 | 3 |
| CN101096 | | TMP-1835 | Fibre | Fibre | Cultivar | Russian Federation | RUS | Central and Eastern Europe | CEE | 82 | 3 |
| CN101099 | | TMP-1838 | Fibre | Fibre | Cultivar | Russian Federation | RUS | Central and Eastern Europe | CEE | 83 | 3 |
| CN101394 | | TMP-13194 | Fibre | Fibre | Unknown | Russian Federation | RUS | Central and Eastern Europe | CEE | 84 | 3 |
| CN101039 | | TMP-1778 | Fibre | Fibre | Breeding line | Russian Federation | RUS | Central and Eastern Europe | CEE | 85 | 3 |
| CN18986 | | TMP-1155 | Fibre | Fibre | Cultivar | France | FRA | Western Europe | WE | 86 | 3 |
| CN18982 | | TMP-1151 | Fibre | Fibre | Cultivar | France | FRA | Western Europe | WE | 87 | 3 |
| CN101094 | | TMP-1833 | Fibre | Fibre | Cultivar | Russian Federation | RUS | Central and Eastern Europe | CEE | 88 | 3 |
| CN101396 | | TMP-13196 | Fibre | Fibre | Unknown | Russian Federation | RUS | Central and Eastern Europe | CEE | 89 | 3 |
| CN101115 | | TMP-1854 | Fibre | Fibre | Breeding line | Russian Federation | RUS | Central and Eastern Europe | CEE | 90 | 3 |
| CN101395 | | TMP-13195 | Fibre | Fibre | Unknown | Russian Federation | RUS | Central and Eastern Europe | CEE | 91 | 3 |
| CN101114 | | TMP-1853 | Fibre | Fibre | Breeding line | Russian Federation | RUS | Central and Eastern Europe | CEE | 92 | 3 |
| CN101600 | | TMP-10024 | Linseed | Linseed | Breeding line | Canada | CAN | North America | NA | 1 | 1 |
| CN100805 | | TMP-1365 | Linseed | Linseed | Cultivar | Czech Republic | CZE | Central and Eastern Europe | CEE | 2 | 1 |
| CN100841 | | TMP-1412 | Linseed | Linseed | Unknown | United Kingdom | UNK | Northern Europe | NE | 3 | 1 |
| CN100799 | | TMP-8366 | Unknown | Linseed | Cultivar | India | IND | Southern Asia | SAS | 4 | 1 |
| CN97129 | | TMP-2124 | Linseed | Linseed | Landrace | Iran | IRN | Southern Asia | SAS | 5 | 1 |
| CN97129B | | TMP-2124-4 | Linseed | Linseed | Landrace | Iran | IRN | Southern Asia | SAS | 6 | 1 |
| CN101338 | | TMP-13137 | Linseed | Linseed | Unknown | Afghanistan | AFG | Southern Asia | SAS | 7 | 1 |
| CN101016 | | TMP-1719 | Unknown | Linseed | Cultivar | China | CHN | Eastern Asia | EA | 8 | 1 |
| CN100837 | | TMP-1408 | Linseed | Linseed | Unknown | Turkey | TUR | Western Asia | WA | 9 | 1 |
| CN97004 | | TMP-8464 | Linseed | Linseed | Cultivar | Ethiopia | ETH | Africa | AF | 10 | 1 |
| CN96991 | | TMP-2087 | Linseed | Linseed | Cultivar | Ethiopia | ETH | Africa | AF | 11 | 1 |
| CN96992 | | TMP-2088 | Linseed | Linseed | Cultivar | Ethiopia | ETH | Africa | AF | 12 | 1 |
| CN100797B | | TMP-8360-6 | Unknown | Linseed | Breeding line | New Zealand | NZL | Oceania | OC | 13 | 2 |
| CN100797 | | TMP-8360-8 | Unknown | Linseed | Breeding line | New Zealand | NZL | Oceania | OC | 14 | 2 |
| CN101598 | | TMP-10126 | Linseed | Linseed | Breeding line | Canada | CAN | North America | NA | 15 | 2 |
| CN100863 | | TMP-1435 | Linseed | Linseed | Breeding line | France | FRA | Western Europe | WE | 16 | 2 |
| CN101451 | | TMP-10033 | Unknown | Linseed | Breeding line | Canada | CAN | North America | NA | 17 | 2 |
| CN101448 | | TMP-10030 | Linseed | Linseed | Breeding line | Canada | CAN | North America | NA | 18 | 2 |
| CN19157 | | TMP-1436 | Linseed | Linseed | Cultivar | Canada | CAN | North America | NA | 19 | 2 |
| CN101454 | | TMP-10109 | Linseed | Linseed | Breeding line | Canada | CAN | North America | NA | 20 | 2 |
| CN100939 | | TMP-1507 | Linseed | Linseed | Cultivar | Russian Federation | RUS | Central and Eastern Europe | CEE | 21 | 2 |
| CN97749 | | TMP-7780 | Linseed | Linseed | Cultivar | United States | USA | North America | NA | 22 | 2 |
| CN97483 | | TMP-7553-6 | Unknown | Linseed | Landrace | Russian Federation | RUS | Central and Eastern Europe | CEE | 23 | 2 |
| CN97605 | | TMP-8376 | Linseed | Linseed | Landrace | Russian Federation | RUS | Central and Eastern Europe | CEE | 24 | 2 |
| CN101331 | | TMP-13130 | Linseed | Linseed | Unknown | Turkey | TUR | Western Asia | WA | 25 | 2 |
| CN97406 | | TMP-2987-14 | Unknown | Linseed | Breeding line | United States | USA | North America | NA | 26 | 2 |
| CN97604 | | TMP-8375 | Linseed | Linseed | Cultivar | Russian Federation | RUS | Central and Eastern Europe | CEE | 27 | 2 |
| CN97489 | | TMP-7559 | Linseed | Linseed | Cultivar | Russian Federation | RUS | Central and Eastern Europe | CEE | 28 | 2 |
| CN97484 | | TMP-7554 | Linseed | Linseed | Cultivar | Russian Federation | RUS | Central and Eastern Europe | CEE | 29 | 2 |
| CN97487 | | TMP-7557 | Linseed | Linseed | Cultivar | Russian Federation | RUS | Central and Eastern Europe | CEE | 30 | 2 |
| CN97403 | | TMP-2984 | Linseed | Linseed | Cultivar | United States | USA | North America | NA | 31 | 2 |
| CN97453 | | TMP-7530 | Unknown | Linseed | Cultivar | United States | USA | North America | NA | 32 | 2 |
| CN100885 | | TMP-1460 | Unknown | Linseed | Unknown | Greece | GRC | Southern Europe | SE | 33 | 2 |
| CN97463 | | TMP-7537 | Linseed | Linseed | Cultivar | United States | USA | North America | NA | 34 | 2 |
| CN97470 | | TMP-10230 | Linseed | Linseed | Cultivar | Japan | JPN | Eastern Asia | EA | 35 | 2 |
| CN97475 | | TMP-7544 | Linseed | Linseed | Cultivar | Russian Federation | RUS | Central and Eastern Europe | CEE | 36 | 2 |
| CN97613 | | TMP-7666 | Linseed | Linseed | Cultivar | Netherlands | NLD | Western Europe | WE | 37 | 2 |
| CN98683 | | TMP-8209 | Linseed | Linseed | Cultivar | Czech Republic | CZE | Central and Eastern Europe | CEE | 38 | 2 |
| CN97886 | | TMP-2361 | Linseed | Linseed | Cultivar | Germany | DEU | Western Europe | WE | 39 | 2 |
| CN97529 | | TMP-7597 | Linseed | Linseed | Cultivar | Russian Federation | RUS | Central and Eastern Europe | CEE | 40 | 2 |
| CN98056 | | TMP-2181 | Linseed | Linseed | Cultivar | Netherlands | NLD | Western Europe | WE | 41 | 2 |
| CN98056B | | TMP-2181-15 | Linseed | Linseed | Cultivar | Netherlands | NLD | Western Europe | WE | 42 | 2 |
| CN97980 | | TMP-2412 | Linseed | Linseed | Cultivar | Argentina | ARG | South America | SA | 43 | 2 |
| CN97350 | | TMP-8235 | Linseed | Linseed | Cultivar | France | FRA | Western Europe | WE | 44 | 2 |
| CN98014 | | TMP-2445 | Linseed | Linseed | Cultivar | Argentina | ARG | South America | SA | 45 | 2 |
| CN98742 | | TMP-8274-8 | Unknown | Linseed | Landrace | France | FRA | Western Europe | WE | 46 | 2 |
| CN101463 | | TMP-10098 | Linseed | Linseed | Breeding line | Canada | CAN | North America | NA | 47 | 2 |
| CN96845 | | TMP-8373 | Linseed | Linseed | Cultivar | Russian Federation | RUS | Central and Eastern Europe | CEE | 48 | 2 |
| CN101469 | | TMP-10112 | Linseed | Linseed | Breeding line | Canada | CAN | North America | NA | 49 | 2 |
| CN101471 | | TMP-9922 | Linseed | Linseed | Breeding line | Canada | CAN | North America | NA | 50 | 2 |
| CN101482 | | TMP-10083 | Linseed | Linseed | Breeding line | Canada | CAN | North America | NA | 51 | 2 |
| CN101560 | | TMP-13679 | Linseed | Linseed | Breeding line | Canada | CAN | North America | NA | 52 | 2 |
| CN97393 | | TMP-2629 | Linseed | Linseed | Cultivar | United States | USA | North America | NA | 53 | 2 |
| CN101472 | | TMP-10082 | Linseed | Linseed | Breeding line | Canada | CAN | North America | NA | 54 | 2 |
| CN96988 | | TMP-2084 | Linseed | Linseed | Cultivar | Ethiopia | ETH | Africa | AF | 55 | 2 |
| CN101307 | | TMP-10896 | Linseed | Linseed | Breeding line | Russian Federation | RUS | Central and Eastern Europe | CEE | 56 | 2 |
| CN101137 | | TMP-8784 | Linseed | Linseed | Breeding line | Russian Federation | RUS | Central and Eastern Europe | CEE | 57 | 2 |
| CN101310 | | TMP-13109 | Linseed | Linseed | Unknown | India | IND | Southern Asia | SAS | 58 | 2 |
| CN98505 | | TMP-8598 | Linseed | Linseed | Cultivar | Russian Federation | RUS | Central and Eastern Europe | CEE | 59 | 2 |
| CN98176 | | TMP-8479 | Linseed | Linseed | Cultivar | Afghanistan | AFG | Southern Asia | SAS | 60 | 2 |
| CN98869 | | TMP-8482 | Linseed | Linseed | Cultivar | Turkey | TUR | Western Asia | WA | 61 | 2 |
| CN100827 | | TMP-1395 | Linseed | Linseed | Cultivar | Slovenia | SVN | Central and Eastern Europe | CEE | 62 | 2 |
| CN96846 | | TMP-8374 | Linseed | Linseed | Cultivar | Russian Federation | RUS | Central and Eastern Europe | CEE | 63 | 2 |
| CN101289 | | TMP-10877 | Linseed | Linseed | Breeding line | Russian Federation | RUS | Central and Eastern Europe | CEE | 64 | 3 |
| CN101241 | | TMP-10817 | Linseed | Linseed | Breeding line | Russian Federation | RUS | Central and Eastern Europe | CEE | 65 | 3 |
| CN101296 | | TMP-10884 | Linseed | Linseed | Breeding line | Russian Federation | RUS | Central and Eastern Europe | CEE | 66 | 3 |
| CN101240 | | TMP-10816 | Linseed | Linseed | Breeding line | Lithuania | LTU | Northern Europe | NE | 67 | 3 |
| CN101237 | | TMP-10812 | Linseed | Linseed | Cultivar | Lithuania | LTU | Northern Europe | NE | 68 | 3 |
| CN101301 | | TMP-10889 | Linseed | Linseed | Breeding line | Russian Federation | RUS | Central and Eastern Europe | CEE | 69 | 3 |
| CDCBethune | | CDC Bethune | Linseed | Linseed | Cultivar | Canada | CAN | North America | NA | 70 | 4 |
| CN33397 | | PGR-5048 | Linseed | Linseed | Cultivar | Canada | CAN | North America | NA | 71 | 4 |
| PrairieBlue | | Prairie Blue | Linseed | Linseed | Cultivar | Canada | CAN | North America | NA | 72 | 4 |
| CN18979 | | TMP-1069 | Linseed | Linseed | Cultivar | Canada | CAN | North America | NA | 73 | 4 |
| CN19007 | | TMP-1440 | Linseed | Linseed | Breeding line | Ethiopia | ETH | Africa | AF | 74 | 4 |
| CDCSorrel | | CDC Sorrel | Linseed | Linseed | Cultivar | Canada | CAN | North America | NA | 75 | 4 |
| CN97873 | | TMP-10239 | Linseed | Linseed | Cultivar | United States | USA | North America | NA | 76 | 4 |
| Shape | | Shape | Linseed | Linseed | Cultivar | Canada | CAN | North America | NA | 77 | 4 |
| Macbeth | | Macbeth | Linseed | Linseed | Cultivar | Canada | CAN | North America | NA | 78 | 4 |
| PrairieThunder | | Prairie Thunder | Linseed | Linseed | Cultivar | Canada | CAN | North America | NA | 79 | 4 |
| CN33992 | | PGR-5772 | Linseed | Linseed | Cultivar | United States | USA | North America | NA | 80 | 4 |
| Lightning | | Lightning | Linseed | Linseed | Cultivar | Canada | CAN | North America | NA | 81 | 4 |
| CN100785 | | TMP-11332 | Linseed | Linseed | Breeding line | United States | USA | North America | NA | 82 | 4 |
| CN18994 | | TMP-1163 | Linseed | Linseed | Cultivar | United States | USA | North America | NA | 83 | 4 |
| CN101610 | | TMP-13685 | Unknown | Linseed | Breeding line | Canada | CAN | North America | NA | 84 | 4 |
| CN100770 | | TMP-11317 | Linseed | Linseed | Breeding line | United States | USA | North America | NA | 85 | 4 |
| CN97334 | | TMP-8148 | Linseed | Linseed | Cultivar | Argentina | ARG | South America | SA | 86 | 4 |
| CN19005 | | TMP-1466 | Linseed | Linseed | Cultivar | Canada | CAN | North America | NA | 87 | 4 |
| CDCMons | | CDC Mons | Linseed | Linseed | Cultivar | Canada | CAN | North America | NA | 88 | 4 |
| CN19003 | | TMP-1310 | Linseed | Linseed | Cultivar | Canada | CAN | North America | NA | 89 | 4 |
| CN97679 | | TMP-7714 | Linseed | Linseed | Breeding line | United States | USA | North America | NA | 90 | 4 |
| CN97679B | | TMP-7714-14 | Linseed | Linseed | Breeding line | United States | USA | North America | NA | 91 | 4 |
| CN101298 | | TMP-10886 | Linseed | Linseed | Breeding line | Russian Federation | RUS | Central and Eastern Europe | CEE | 92 | 4 |
| CN101367 | | TMP-13166 | Linseed | Linseed | Unknown | Georgia | GEO | Western Asia | WA | 93 | 4 |
| CN101299 | | TMP-10887 | Linseed | Linseed | Breeding line | Russian Federation | RUS | Central and Eastern Europe | CEE | 94 | 4 |
| CN97639 | | TMP-7684 | Linseed | Linseed | Cultivar | United States | USA | North America | NA | 95 | 4 |
| CN97639B | | TMP-7684-11 | Linseed | Linseed | Cultivar | United States | USA | North America | NA | 96 | 4 |
| CN97404 | | TMP-2985 | Linseed | Linseed | Breeding line | United States | USA | North America | NA | 97 | 4 |
| CN97404B | | TMP-2985-5 | Linseed | Linseed | Breeding line | United States | USA | North America | NA | 98 | 4 |
| CN97642 | | TMP-7687 | Linseed | Linseed | Cultivar | United States | USA | North America | NA | 99 | 4 |
| CN97402 | | TMP-2983-13 | Unknown | Linseed | Breeding line | United States | USA | North America | NA | 100 | 4 |
| CN101286 | | TMP-10873 | Linseed | Linseed | Breeding line | United States | USA | North America | NA | 101 | 4 |
| CN97458 | | TMP-2322 | Linseed | Linseed | Cultivar | Netherlands | NLD | Western Europe | WE | 102 | 4 |
| CN97377 | | TMP-2968 | Linseed | Linseed | Cultivar | United States | USA | North America | NA | 103 | 4 |
| CN97397 | | TMP-2979 | Unknown | Linseed | Cultivar | United States | USA | North America | NA | 104 | 4 |
| CN101308 | | TMP-13107 | Linseed | Linseed | Unknown | India | IND | Southern Asia | SAS | 105 | 4 |
| CN101329 | | TMP-13128 | Linseed | Linseed | Unknown | Egypt | EGY | Africa | AF | 106 | 4 |
| CN101332 | | TMP-13131 | Linseed | Linseed | Unknown | Turkey | TUR | Western Asia | WA | 107 | 4 |
| CN101325 | | TMP-13124 | Linseed | Linseed | Unknown | Greece | GRC | Southern Europe | SE | 108 | 4 |
| CN30860 | | PGR-1725 | Linseed | Linseed | Cultivar | Ukraine | UKR | Central and Eastern Europe | CEE | 109 | 4 |
| CN97587 | | TMP-7649 | Linseed | Linseed | Cultivar | United States | USA | North America | NA | 110 | 4 |
| CN37286 | | PGR-10014 | Linseed | Linseed | Cultivar | Canada | CAN | North America | NA | 111 | 4 |
| CN33388 | | PGR-5039 | Linseed | Linseed | Cultivar | Canada | CAN | North America | NA | 112 | 4 |
| Hanley | | Hanley | Linseed | Linseed | Cultivar | Canada | CAN | North America | NA | 113 | 4 |
| CN97649 | | TMP-7694 | Linseed | Linseed | Cultivar | United States | USA | North America | NA | 114 | 4 |
| CN33400 | | PGR-5051 | Linseed | Linseed | Cultivar | United States | USA | North America | NA | 115 | 4 |
| CN19004 | | TMP-1311 | Linseed | Linseed | Cultivar | Canada | CAN | North America | NA | 116 | 4 |
| CN33386 | | PGR-5037 | Linseed | Linseed | Cultivar | Canada | CAN | North America | NA | 117 | 4 |
| CN33389 | | PGR-5040 | Linseed | Linseed | Cultivar | Canada | CAN | North America | NA | 118 | 4 |
| CN18989 | | TMP-1158 | Linseed | Linseed | Cultivar | France | FRA | Western Europe | WE | 119 | 4 |
| CN98773 | | TMP-8307 | Linseed | Linseed | Cultivar | France | FRA | Western Europe | WE | 120 | 4 |
| CN97586 | | TMP-7648 | Linseed | Linseed | Cultivar | United States | USA | North America | NA | 121 | 4 |
| CN98812 | | TMP-8345 | Linseed | Linseed | Cultivar | United States | USA | North America | NA | 122 | 4 |
| CN97728 | | TMP-7760 | Linseed | Linseed | Cultivar | United States | USA | North America | NA | 123 | 4 |
| CN97881 | | TMP-7880 | Linseed | Linseed | Cultivar | United States | USA | North America | NA | 124 | 4 |
| CN19160 | | TMP-1384 | Linseed | Linseed | Cultivar | United States | USA | North America | NA | 125 | 4 |
| CN97214 | | TMP-2564 | Linseed | Linseed | Cultivar | Argentina | ARG | South America | SA | 126 | 4 |
| CN19158 | | TMP-1508 | Linseed | Linseed | Cultivar | Canada | CAN | North America | NA | 127 | 4 |
| CN100807 | | TMP-1368 | Linseed | Linseed | Breeding line | Afghanistan | AFG | Southern Asia | SAS | 128 | 4 |
| CN101265 | | TMP-10847 | Linseed | Linseed | Cultivar | Great Britain | GBR | Northern Europe | NE | 129 | 4 |
| CN100884 | | TMP-1459 | Linseed | Linseed | Cultivar | Czechoslovakia | CSK | Central and Eastern Europe | CEE | 130 | 4 |
| CN18980 | | TMP-1070 | Linseed | Linseed | Cultivar | Canada | CAN | North America | NA | 131 | 4 |
| CN18981 | | TMP-1097 | Linseed | Linseed | Cultivar | Canada | CAN | North America | NA | 132 | 4 |
| CN52732 | | PGR-27314 | Linseed | Linseed | Cultivar | Canada | CAN | North America | NA | 133 | 4 |
| CN19017 | | TMP-8663 | Linseed | Linseed | Cultivar | Canada | CAN | North America | NA | 134 | 4 |
| CN97584 | | TMP-7646 | Unknown | Linseed | Breeding line | United States | USA | North America | NA | 135 | 4 |
| CN97584B | | TMP-7646-7 | Unknown | Linseed | Breeding line | United States | USA | North America | NA | 136 | 4 |
| CN97671 | | TMP-2173 | Linseed | Linseed | Cultivar | Canada | CAN | North America | NA | 137 | 4 |
| CN97520 | | TMP-7590 | Linseed | Linseed | Cultivar | Russian Federation | RUS | Central and Eastern Europe | CEE | 138 | 4 |
| PrairieGrande | | Prairie Grande | Linseed | Linseed | Cultivar | Canada | CAN | North America | NA | 139 | 4 |
| CN18973 | | TMP-605 | Linseed | Linseed | Cultivar | Canada | CAN | North America | NA | 140 | 4 |
| CN101132 | | TMP-1871 | Linseed | Linseed | Breeding line | Russian Federation | RUS | Central and Eastern Europe | CEE | 141 | 4 |
| CN30861 | | PGR-1726 | Linseed | Linseed | Cultivar | United Kingdom | UNK | Northern Europe | NE | 142 | 4 |
| CN101413 | | TMP-1174 | Linseed | Linseed | Cultivar | Canada | CAN | North America | NA | 143 | 4 |
| CN33399 | | PGR-5050 | Linseed | Linseed | Cultivar | United States | USA | North America | NA | 144 | 4 |
| CN97740 | | TMP-7771 | Linseed | Linseed | Cultivar | United States | USA | North America | NA | 145 | 4 |
| CN100547 | | PGR-14271 | Linseed | Linseed | Cultivar | United Kingdom | UNK | Northern Europe | NE | 146 | 4 |
| CN97444 | | TMP-7521 | Linseed | Linseed | Cultivar | United States | USA | North America | NA | 147 | 4 |
| CN33385 | | PGR-5036 | Linseed | Linseed | Cultivar | Canada | CAN | North America | NA | 148 | 4 |
| CN97392 | | TMP-2975 | Linseed | Linseed | Cultivar | Canada | CAN | North America | NA | 149 | 4 |
| Linola989 | | TMP-14331 | Linseed | Linseed | Cultivar | Canada | CAN | North America | NA | 150 | 5 |
| CDCGold | | TMP-17618 | Linseed | Linseed | Cultivar | Canada | CAN | North America | NA | 151 | 5 |
| CN101373 | | TMP-13173 | Linseed | Linseed | Unknown | Armenia | ARM | Western Asia | WA | 152 | 6 |
| CN98231 | | TMP-8533 | Linseed | Linseed | Cultivar | United States | USA | North America | NA | 153 | 6 |
| CN98239 | | TMP-8535 | Linseed | Linseed | Cultivar | Pakistan | PAK | Southern Asia | SAS | 154 | 6 |
| CN100629 | | TMP-11172 | Linseed | Linseed | Cultivar | Pakistan | PAK | Southern Asia | SAS | 155 | 6 |
| CN100790 | | TMP-11337 | Unknown | Linseed | Cultivar | Pakistan | PAK | Southern Asia | SAS | 156 | 6 |
| CN98007 | | TMP-2438 | Linseed | Linseed | Cultivar | Argentina | ARG | South America | SA | 157 | 6 |
| CN97768 | | TMP-2343 | Unknown | Linseed | Cultivar | Portugal | PRT | Southern Europe | SE | 158 | 6 |
| CN97953 | | TMP-2386 | Linseed | Linseed | Cultivar | Argentina | ARG | South America | SA | 159 | 6 |
| CN98613 | | TMP-2963 | Linseed | Linseed | Cultivar | United States | USA | North America | NA | 160 | 6 |
| CN98254 | | TMP-8018 | Linseed | Linseed | Cultivar | India | IND | Southern Asia | SAS | 161 | 6 |
| CN97103 | | TMP-8560 | Linseed | Linseed | Cultivar | Pakistan | PAK | Southern Asia | SAS | 162 | 6 |
| CN98821 | | TMP-8123 | Linseed | Linseed | Cultivar | United States | USA | North America | NA | 163 | 6 |
| CN97096 | | TMP-2756 | Linseed | Linseed | Cultivar | Pakistan | PAK | Southern Asia | SAS | 164 | 6 |
| CN97092 | | TMP-8556 | Linseed | Linseed | Cultivar | Pakistan | PAK | Southern Asia | SAS | 165 | 6 |
| CN98037 | | TMP-2467 | Linseed | Linseed | Breeding line | Argentina | ARG | South America | SA | 166 | 6 |
| CN98037B | | TMP-2467-8 | Linseed | Linseed | Breeding line | Argentina | ARG | South America | SA | 167 | 6 |
| CN101366 | | TMP-13165 | Linseed | Linseed | Unknown | Georgia | GEO | Western Asia | WA | 168 | 6 |
| CN101375 | | TMP-13175 | Linseed | Linseed | Unknown | Russian Federation | RUS | Central and Eastern Europe | CEE | 169 | 6 |
| CN97961 | | TMP-2393 | Linseed | Linseed | Cultivar | Argentina | ARG | South America | SA | 170 | 6 |
| CN97300 | | TMP-8073 | Linseed | Linseed | Cultivar | Hungary | HUN | Central and Eastern Europe | CEE | 171 | 6 |
| CN96958 | | TMP-2650-14 | Linseed | Linseed | Landrace | Turkey | TUR | Western Asia | WA | 172 | 6 |
| CN101493 | | TMP-9941 | Linseed | Linseed | Breeding line | Canada | CAN | North America | NA | 173 | 6 |
| CN18993 | | TMP-1162 | Linseed | Linseed | Cultivar | Netherlands | NLD | Western Europe | WE | 174 | 6 |
| CN101536 | | TMP-9981 | Linseed | Linseed | Breeding line | Canada | CAN | North America | NA | 175 | 6 |
| CN97341 | | TMP-8152-12 | Unknown | Linseed | Cultivar | Argentina | ARG | South America | SA | 176 | 6 |
| CN98027 | | TMP-2457 | Linseed | Linseed | Cultivar | Argentina | ARG | South America | SA | 177 | 6 |
| CN98012 | | TMP-2443 | Linseed | Linseed | Cultivar | Argentina | ARG | South America | SA | 178 | 6 |
| CN98794 | | TMP-8329 | Linseed | Linseed | Cultivar | France | FRA | Western Europe | WE | 179 | 6 |
| CN101596 | | TMP-10021 | Linseed | Linseed | Breeding line | Canada | CAN | North America | NA | 180 | 6 |
| CN98807 | | TMP-8340 | Linseed | Linseed | Cultivar | France | FRA | Western Europe | WE | 181 | 6 |
| CN97366 | | TMP-8641 | Linseed | Linseed | Cultivar | United States | USA | North America | NA | 182 | 6 |
| CN101554 | | TMP-9996 | Linseed | Linseed | Breeding line | Canada | CAN | North America | NA | 183 | 6 |
| CN97907 | | TMP-7899 | Linseed | Linseed | Cultivar | United States | USA | North America | NA | 184 | 6 |
| CN98806 | | TMP-8339 | Linseed | Linseed | Breeding line | France | FRA | Western Europe | WE | 185 | 6 |
| CN98542 | | TMP-2622 | Linseed | Linseed | Cultivar | United States | USA | North America | NA | 186 | 6 |
| CN97890 | | TMP-8382 | Linseed | Linseed | Cultivar | United States | USA | North America | NA | 187 | 6 |
| CN98752 | | TMP-8284 | Linseed | Linseed | Cultivar | France | FRA | Western Europe | WE | 188 | 6 |
| CN98753 | | TMP-8285 | Linseed | Linseed | Cultivar | France | FRA | Western Europe | WE | 189 | 6 |
| CN96962 | | TMP-8451 | Linseed | Linseed | Cultivar | Turkey | TUR | Western Asia | WA | 190 | 6 |
| CN97718 | | TMP-7751 | Linseed | Linseed | Cultivar | United States | USA | North America | NA | 191 | 6 |
| CN97571 | | TMP-7636 | Linseed | Linseed | Cultivar | Canada | CAN | North America | NA | 192 | 6 |
| CN98741 | | TMP-8273-2 | Linseed | Linseed | Breeding line | France | FRA | Western Europe | WE | 193 | 6 |
| CN98733 | | TMP-8267-8 | Linseed | Linseed | Breeding line | Poland | POL | Central and Eastern Europe | CEE | 194 | 6 |
| CN96911 | | TMP-8409 | Linseed | Linseed | Cultivar | Turkey | TUR | Western Asia | WA | 195 | 6 |
| CN97153 | | TMP-8574 | Linseed | Linseed | Cultivar | Turkey | TUR | Western Asia | WA | 196 | 6 |
| CN98100 | | TMP-2508 | Linseed | Linseed | Cultivar | Uruguay | URY | South America | SA | 197 | 6 |
| CN97147 | | TMP-8570 | Linseed | Linseed | Cultivar | Turkey | TUR | Western Asia | WA | 198 | 6 |
| CN98165 | | TMP-7982 | Linseed | Linseed | Cultivar | Iran | IRN | Southern Asia | SAS | 199 | 6 |
| CN101594 | | TMP-10125 | Linseed | Linseed | Breeding line | Canada | CAN | North America | NA | 200 | 6 |
| CN100828 | | TMP-1396 | Unknown | Linseed | Unknown | Turkey | TUR | Western Asia | WA | 201 | 6 |
| CN101279 | | TMP-10862 | Linseed | Linseed | Breeding line | Russian Federation | RUS | Central and Eastern Europe | CEE | 202 | 6 |
| CN98734 | | TMP-8268 | Linseed | Linseed | Cultivar | France | FRA | Western Europe | WE | 203 | 6 |
| CN97430 | | TMP-2998 | Linseed | Linseed | Breeding line | Germany | DEU | Western Europe | WE | 204 | 6 |
| CN97430B | | TMP-2998-9 | Linseed | Linseed | Breeding line | Germany | DEU | Western Europe | WE | 205 | 6 |
| CN101026 | | TMP-1729 | Linseed | Linseed | Breeding line | Morocco | MAR | Africa | AF | 206 | 6 |
| CN98275 | | TMP-2535 | Linseed | Linseed | Cultivar | Hungary | HUN | Central and Eastern Europe | CEE | 207 | 6 |
| CN97238 | | TMP-2932 | Linseed | Linseed | Cultivar | Hungary | HUN | Central and Eastern Europe | CEE | 208 | 6 |
| CN98039 | | TMP-2469 | Linseed | Linseed | Cultivar | Argentina | ARG | South America | SA | 209 | 6 |
| CN98644 | | TMP-8168-3 | Unknown | Linseed | Breeding line | United States | USA | North America | NA | 210 | 6 |
| CN97670 | | TMP-7706-13 | Unknown | Linseed | Breeding line | United States | USA | North America | NA | 211 | 6 |
| CN98278 | | TMP-2538 | Linseed | Linseed | Cultivar | Hungary | HUN | Central and Eastern Europe | CEE | 212 | 6 |
| CN97689 | | TMP-7723 | Linseed | Linseed | Cultivar | United States | USA | North America | NA | 213 | 6 |
| CN100674 | | TMP-11220 | Linseed | Linseed | Cultivar | Romania | ROM | Central and Eastern Europe | CEE | 214 | 6 |
| CN97287 | | TMP-2610 | Linseed | Linseed | Cultivar | Hungary | HUN | Central and Eastern Europe | CEE | 215 | 6 |
| CN97321 | | TMP-8126 | Linseed | Linseed | Cultivar | Romania | ROM | Central and Eastern Europe | CEE | 216 | 6 |
| CN98279 | | TMP-10221 | Linseed | Linseed | Cultivar | Argentina | ARG | South America | SA | 217 | 6 |
| CN97967 | | TMP-2399 | Linseed | Linseed | Cultivar | Argentina | ARG | South America | SA | 218 | 6 |
| CN19159 | | TMP-1354 | Linseed | Linseed | Cultivar | Canada | CAN | North America | NA | 219 | 6 |
| CN97407 | | TMP-2988 | Linseed | Linseed | Cultivar | United States | USA | North America | NA | 220 | 6 |
| CN101461 | | TMP-10097 | Linseed | Linseed | Breeding line | Canada | CAN | North America | NA | 221 | 6 |
| CN101565 | | TMP-10004 | Linseed | Linseed | Breeding line | Canada | CAN | North America | NA | 222 | 6 |
| CN98475 | | TMP-2279 | Linseed | Linseed | Cultivar | Germany | DEU | Western Europe | WE | 223 | 6 |
| CN101208 | | TMP-9853 | Linseed | Linseed | Cultivar | India | IND | Southern Asia | SAS | 224 | 6 |
| CN97921 | | TMP-7912 | Linseed | Linseed | Cultivar | United States | USA | North America | NA | 225 | 6 |
| CN100851 | | TMP-1422 | Linseed | Linseed | Cultivar | Czech Republic | CZE | Central and Eastern Europe | CEE | 226 | 6 |
| CN98639 | | TMP-8163 | Linseed | Linseed | Cultivar | United States | USA | North America | NA | 227 | 6 |
| CN98712 | | TMP-8240 | Linseed | Linseed | Cultivar | France | FRA | Western Europe | WE | 228 | 6 |
| CN101595 | | TMP-10020 | Linseed | Linseed | Breeding line | Canada | CAN | North America | NA | 229 | 6 |
| CN100883 | | TMP-1457 | Linseed | Linseed | Cultivar | Hungary | HUN | Central and Eastern Europe | CEE | 230 | 6 |
| CN101496 | | TMP-13674 | Linseed | Linseed | Breeding line | Canada | CAN | North America | NA | 231 | 6 |
| CN100881 | | TMP-1455 | Linseed | Linseed | Cultivar | Germany | DEU | Western Europe | WE | 232 | 6 |
| CN98193 | | TMP-2530 | Unknown | Linseed | Cultivar | Morocco | MAR | Africa | AF | 233 | 6 |
| CN100838 | | TMP-1409 | Linseed | Linseed | Unknown | Cyprus | CYP | Western Asia | WA | 234 | 6 |
| CN97958 | | TMP-2390 | Linseed | Linseed | Cultivar | Argentina | ARG | South America | SA | 235 | 6 |
| CN98689 | | TMP-8216-5 | Unknown | Linseed | Cultivar | Czech Republic | CZE | Central and Eastern Europe | CEE | 236 | 6 |
| CN97633 | | TMP-7678 | Linseed | Linseed | Cultivar | Canada | CAN | North America | NA | 237 | 6 |
| CN98634 | | TMP-8158 | Linseed | Linseed | Cultivar | Argentina | ARG | South America | SA | 238 | 6 |
| CN98984 | | TMP-8172 | Linseed | Linseed | Cultivar | Australia | AUS | Oceania | OC | 239 | 6 |
| CN98767 | | TMP-8301 | Linseed | Linseed | Cultivar | France | FRA | Western Europe | WE | 240 | 6 |
| CN100928 | | TMP-1613 | Linseed | Linseed | Cultivar | France | FRA | Western Europe | WE | 241 | 6 |
| CN98276 | | TMP-2536 | Linseed | Linseed | Cultivar | Hungary | HUN | Central and Eastern Europe | CEE | 242 | 6 |
| CN98370 | | TMP-2817 | Linseed | Linseed | Cultivar | India | IND | Southern Asia | SAS | 243 | 7 |
| CN97308 | | TMP-2940 | Linseed | Linseed | Cultivar | India | IND | Southern Asia | SAS | 244 | 7 |
| CN101539 | | TMP-9982 | Linseed | Linseed | Breeding line | Canada | CAN | North America | NA | 245 | 7 |
| CN98569 | | TMP-2953 | Linseed | Linseed | Unknown | India | IND | Southern Asia | SAS | 246 | 7 |
| CN97306 | | TMP-2938 | Linseed | Linseed | Cultivar | India | IND | Southern Asia | SAS | 247 | 7 |
| CN98157 | | TMP-7973 | Linseed | Linseed | Cultivar | India | IND | Southern Asia | SAS | 248 | 7 |
| CN98192 | | TMP-8006 | Linseed | Linseed | Cultivar | Ireland | IRL | Northern Europe | NE | 249 | 7 |
| CN98566C | | TMP-2945-4 | Linseed | Linseed | Breeding line | United States | USA | North America | NA | 250 | 7 |
| CN98566 | | TMP-2945 | Linseed | Linseed | Breeding line | United States | USA | North America | NA | 251 | 7 |
| CN98566B | | TMP-2945-7 | Linseed | Linseed | Breeding line | United States | USA | North America | NA | 252 | 7 |
| CN97396 | | TMP-2978 | Linseed | Linseed | Cultivar | United States | USA | North America | NA | 253 | 8 |
| CN100852 | | TMP-1423 | Unknown | Linseed | Cultivar | Portugal | PRT | Southern Europe | SE | 254 | 8 |
| CN100910 | | TMP-1595 | Linseed | Linseed | Landrace | Portugal | PRT | Southern Europe | SE | 255 | 8 |
| CN96974 | | TMP-2652-15 | Linseed | Linseed | Landrace | India | IND | Southern Asia | SAS | 256 | 8 |
| CN97064 | | TMP-2705 | Linseed | Linseed | Cultivar | Pakistan | PAK | Southern Asia | SAS | 257 | 8 |
| CN97056 | | TMP-2697 | Linseed | Linseed | Cultivar | Pakistan | PAK | Southern Asia | SAS | 258 | 8 |
| CN98363 | | TMP-2810 | Linseed | Linseed | Cultivar | India | IND | Southern Asia | SAS | 259 | 8 |
| CN98237 | | TMP-2677 | Linseed | Linseed | Cultivar | Pakistan | PAK | Southern Asia | SAS | 260 | 8 |
| CN101535 | | TMP-9980 | Linseed | Linseed | Breeding line | Canada | CAN | North America | NA | 261 | 8 |
| CN98263B | | TMP-2534-5 | Linseed | Linseed | Cultivar | Hungary | HUN | Central and Eastern Europe | CEE | 262 | 8 |
| CN98263 | | TMP-2534 | Linseed | Linseed | Cultivar | Hungary | HUN | Central and Eastern Europe | CEE | 263 | 8 |
| CN101511 | | TMP-9953 | Linseed | Linseed | Breeding line | Canada | CAN | North America | NA | 264 | 8 |
| CN98541 | | TMP-2934 | Linseed | Linseed | Cultivar | United States | USA | North America | NA | 265 | 8 |
| CN98250 | | TMP-8017 | Linseed | Linseed | Cultivar | India | IND | Southern Asia | SAS | 266 | 8 |
| CN101466 | | TMP-10135 | Linseed | Linseed | Breeding line | Canada | CAN | North America | NA | 267 | 8 |
| CN100678 | | TMP-11224 | Unknown | Linseed | Cultivar | Romania | ROM | Central and Eastern Europe | CEE | 268 | 8 |
| CN101510 | | TMP-9952 | Linseed | Linseed | Breeding line | Canada | CAN | North America | NA | 269 | 8 |
| CN97050 | | TMP-2695 | Linseed | Linseed | Cultivar | Iran | IRN | Southern Asia | SAS | 270 | 8 |
| CN98057 | | TMP-2655 | Linseed | Linseed | Cultivar | India | IND | Southern Asia | SAS | 271 | 8 |
| CN97083 | | TMP-2724-10 | Linseed | Linseed | Landrace | Pakistan | PAK | Southern Asia | SAS | 272 | 8 |
| CN98240 | | TMP-2679-15 | Linseed | Linseed | Landrace | India | IND | Southern Asia | SAS | 273 | 8 |
| CN98974 | | TMP-2895 | Linseed | Linseed | Cultivar | India | IND | Southern Asia | SAS | 274 | 8 |
| CN97139 | | TMP-2772 | Linseed | Linseed | Cultivar | Iran | IRN | Southern Asia | SAS | 275 | 8 |
| CN98135 | | TMP-2671 | Linseed | Linseed | Cultivar | India | IND | Southern Asia | SAS | 276 | 8 |
| CN98468 | | TMP-2924 | Linseed | Linseed | Cultivar | India | IND | Southern Asia | SAS | 277 | 8 |
| CN98440 | | TMP-2887 | Linseed | Linseed | Cultivar | India | IND | Southern Asia | SAS | 278 | 8 |
| CN101542 | | TMP-9984 | Linseed | Linseed | Breeding line | Canada | CAN | North America | NA | 279 | 8 |
| CN98982 | | TMP-2922 | Linseed | Linseed | Cultivar | India | IND | Southern Asia | SAS | 280 | 8 |
| CN98240B | | TMP-2679-9 | Linseed | Linseed | Landrace | India | IND | Southern Asia | SAS | 281 | 8 |
| CN98364 | | TMP-2811 | Linseed | Linseed | Cultivar | India | IND | Southern Asia | SAS | 282 | 8 |
| CN98242 | | TMP-2681-1 | Linseed | Linseed | Landrace | India | IND | Southern Asia | SAS | 283 | 8 |
| CN98397 | | TMP-2844 | Linseed | Linseed | Cultivar | India | IND | Southern Asia | SAS | 284 | 8 |
| CN98109 | | TMP-2659 | Linseed | Linseed | Cultivar | India | IND | Southern Asia | SAS | 285 | 8 |
| CN98398 | | TMP-2845 | Linseed | Linseed | Cultivar | India | IND | Southern Asia | SAS | 286 | 8 |
| CN97312 | | TMP-2948 | Linseed | Linseed | Cultivar | India | IND | Southern Asia | SAS | 287 | 8 |
| CN98854 | | TMP-10222 | Linseed | Linseed | Cultivar | Hungary | HUN | Central and Eastern Europe | CEE | 288 | 8 |
| CN97176 | | TMP-2280 | Linseed | Linseed | Cultivar | Czech Republic | CZE | Central and Eastern Europe | CEE | 289 | 8 |
| CN97072 | | TMP-2713-4 | Linseed | Linseed | Landrace | Pakistan | PAK | Southern Asia | SAS | 290 | 8 |
| CN101580 | | TMP-10121 | Linseed | Linseed | Breeding line | Canada | CAN | North America | NA | 291 | 8 |
| CN98610 | | TMP-2960 | Unknown | Linseed | Cultivar | United States | USA | North America | NA | 292 | 8 |
| CN97307 | | TMP-2939 | Linseed | Linseed | Cultivar | India | IND | Southern Asia | SAS | 293 | 8 |
| CN98961 | | TMP-8020 | Linseed | Linseed | Cultivar | India | IND | Southern Asia | SAS | 294 | 8 |
| CN98535 | | TMP-8643 | Linseed | Linseed | Cultivar | United States | USA | North America | NA | 295 | 8 |
| CN98969 | | TMP-2795 | Linseed | Linseed | Cultivar | India | IND | Southern Asia | SAS | 296 | 8 |
| CN98973 | | TMP-2894 | Linseed | Linseed | Cultivar | India | IND | Southern Asia | SAS | 297 | 8 |
| CN98415 | | TMP-2862 | Linseed | Linseed | Cultivar | India | IND | Southern Asia | SAS | 298 | 8 |
| CN98467 | | TMP-2923 | Linseed | Linseed | Cultivar | India | IND | Southern Asia | SAS | 299 | 8 |

abbrev.: abbreviation
