# Supplementary material for: Genetic Variability of 27 Traits in a Core Collection of Flax (Linum usitatissimum L.)
Source: Front Plant Sci. 2017 Sep 21;8:1636. doi: 10.3389/fpls.2017.01636 (PMC5622609; doi:10.3389/fpls.2017.01636)
Supplement: Supplementary file 3 [file Table3.DOCX]

**TABLE S3** Joint analysis of variance of genotype, year, location and their interactions for 27 traits.

| **Trait** | **Abbreviation** | **Mean square and significance** | | | | | | |
| --- | --- | --- | --- | --- | --- | --- | --- | --- |
|  |  | **Genotype (G)** | **Location (L)** | **Year (Y)** | **G×Y** | **G×Y** | **L×Y** | **G×L×Y** |
| Seed yield (t·ha^-1^) | YLD | 94.4*** | 8242*** | 16627.3*** | 9.10 | 16.10 | 21014.3*** | 11.70 |
| Seeds boll^-1^ | SEB | 5.521005*** | 1966.74*** | 28.34597*** | 1.17399703** | 1.67*** | 16.03*** | 0.98 |
| Seeds m^-^² | SM2 | 77247500*** | 3852811000*** | 88964620* | 13936970.00 | 18142910*** | 287055700*** | 14848050.00 |
| Thousand-seed weight (g) | TSW | 59.35*** | NA | 214.04*** | NA | 0.32*** | NA | NA |
| Bolls m^-^² | BM2 | 997985.3*** | 12717160*** | 9635923*** | 245753.90 | 286251.7** | 13443980*** | 262511.4* |
| Lodging | LOD | 1.30*** | 35.10*** | 1.12 | 0.80 | 0.53 | 49.78*** | 0.58 |
| Days to flowering | DTF | 67.30*** | 7712.62*** | 9317.83*** | 4.47 | 5.13 | 227.77*** | 4.07 |
| Days to maturity | DTM | 113.83*** | 42175.66*** | 88919.04*** | 37.04 | 44.02 | 29981.75*** | 14.98 |
| Plant height (cm) | PLH | 1124.40*** | 421016.2*** | 135520.6*** | 116.1702*** | 102.63* | 36801.75*** | 79.90 |
| Branching score | BSC | 1.34 | 2169.33*** | 38.06*** | 2.81*** | 1.32 | 0.10 | 1.05 |
| Protein content (%) | PRO | 12.9*** | 9848*** | 12.2*** | 3.1*** | 1.3*** | 2169*** | 0.6*** |
| Linseed content (%) | OIL | 411568.00 | 395459.00 | 90649.00 | 411856.00 | 83469.00 | 61285.00 | 83869.00 |
| Iodine value | IOD | 394.90*** | 108808.8*** | 7082.08*** | 16.25 | 30.77 | 8.49 | 10.74 |
| Palmitic (%) | PAL | 3.11*** | 8.2708*** | 17.50*** | 0.1182** | 0.1386*** | 3.37*** | 0.06 |
| Stearic (%) | STE | 8.49*** | 478.69*** | 66.51*** | 0.31*** | 0.52*** | 41.37*** | 0.13 |
| Oleic (%) | OLE | 75.5*** | 28330.5*** | 1739.1*** | 3.90 | 7.20 | 13.50 | 2.70 |
| Linoleic (%) | LIO | 136.80*** | 1221.77*** | 3.93771119* | 1.03144387** | 5.39*** | 105.62*** | 0.63 |
| Linolenic (%) | LIN | 412912.50 | 540398.10 | 91162.36 | 412969.00 | 82735.07 | 71954.06 | 84075.44 |
| Straw weight (g) | STR | 497*** | 184212*** | 1.00 | 427*** | 16.00 | NA | NA |
| Fibre (%) | FIB | 32.49*** | 36453.65*** | 25867.27*** | 10.01 | 9.90 | 18338.8*** | 6.77 |
| Lignin (%) | LIG | 0.67*** | 648.95*** | 539.60*** | 0.21 | 0.21 | 417.38*** | 0.14 |
| Shive (%) | SHI | 33*** | 43928*** | 26412*** | 10.00 | 10.00 | 19077*** | 7.00 |
| Cell walls (%) | CEW | 7.03*** | 5059.61*** | 4401.525*** | 3.22 | 2.89 | 1120.17*** | 3.04 |
| Cellulose (%) | CEL | 31.36*** | 58455.73*** | 36244.82*** | 9.02 | 10.50 | 26413.17*** | 7.82 |
| Pasmo score | PAS | 4.5*** | 2168.5*** | 5916.2*** | 0.90 | 2.5** | 10.4* | 0.70 |
| Powdery mildew score | MIL | 7.15*** | NA | 677.97*** | NA | 1.55 | NA | NA |
| Fusarium wilt score | WIL | 3.33*** | NA | NA | NA | NA | NA | NA |

*, **, and *** represent statistical significance at 0.05, 0.01 and 0.001 probability levels, respectively.
