# Supplementary material for: Genetic Variability of 27 Traits in a Core Collection of Flax (Linum usitatissimum L.)
Source: Front Plant Sci. 2017 Sep 21;8:1636. doi: 10.3389/fpls.2017.01636 (PMC5622609; doi:10.3389/fpls.2017.01636)
Supplement: Supplementary file 4 [file Table4.DOCX]

**TABLE S4** Phenotypic variance partitioning of 27 traits in terms of morphotype, geographic origin and other factors for 391 accessions of the core collection.

| **Trait** | **Abbreviation** | **Morphotype (%)** | **Geographical region (%)** | **Other (%)** |
| --- | --- | --- | --- | --- |
| Seed yield (t·ha^-1^) | YLD | 8.39 | 28.45 | 63.16 |
| Seeds boll^-1^ | SEB | 0.00 | 11.51 | 88.49 |
| Seeds m^-^² | SM2 | 5.81 | 12.01 | 82.19 |
| Thousand-seed weight (g) | TSW | 25.17 | 0.57 | 74.27 |
| Bolls m^-^² | BM2 | 6.10 | 16.25 | 77.65 |
| Lodging | LOD | 6.83 | 18.11 | 75.07 |
| Days to flowering | DTF | 9.30 | 38.36 | 52.34 |
| Days to maturity | DTM | 7.31 | 12.86 | 79.83 |
| Plant height (cm) | PLH | 64.34 | 11.66 | 24.00 |
| Branching score | BSC | 29.34 | 2.98 | 67.68 |
| Protein content (%) | PRO | 67.60 | 1.40 | 31.00 |
| Oil content (%) | OIL | 45.24 | 10.39 | 44.37 |
| Iodine (%) | IOD | 0.77 | 9.64 | 89.59 |
| Palmitic (%) | PAL | 61.23 | 1.69 | 37.07 |
| Stearic (%) | STE | 0.00 | 15.06 | 84.93 |
| Oleic (%) | OLE | 0.00 | 11.14 | 88.86 |
| Linoleic (%) | LIO | 5.62 | 3.23 | 91.15 |
| Linolenic (%) | LIN | 0.00 | 0.40 | 99.59 |
| Straw weight (g) | STR | 60.34 | 14.35 | 25.31 |
| Fibre (%) | FIB | 23.52 | 13.67 | 62.81 |
| Lignin (%) | LIG | 23.07 | 13.80 | 63.13 |
| Shive (%) | SHI | 23.87 | 13.58 | 62.55 |
| Cell walls (%) | CEW | 21.26 | 6.50 | 72.24 |
| Cellulose (%) | CEL | 11.37 | 9.14 | 79.49 |
| Pasmo score | PAS | 43.83 | 11.07 | 45.09 |
| Powdery mildew score | MIL | 27.68 | 0.10 | 72.22 |
| Fusarium wilt score | WIL | 16.07 | 8.34 | 75.59 |
| Average |  | 22.00 | 10.97 | 67.03 |
