# Supplementary material for: Genetic Variability of 27 Traits in a Core Collection of Flax (Linum usitatissimum L.)
Source: Front Plant Sci. 2017 Sep 21;8:1636. doi: 10.3389/fpls.2017.01636 (PMC5622609; doi:10.3389/fpls.2017.01636)
Supplement: Supplementary file 5 [file Table5.DOCX]

**TABLE S5** Linear discrimination function for morphotype using 27 traits. The numeric values represent coefficients of variables (constant term and traits).

| **Variable** | **Abbreviation** | **Fibre** | **Linseed** |
| --- | --- | --- | --- |
| Constant |  | -2.9E+07 | -2.9E+07 |
| Seed yield (t·ha^-1^) | YLD | 281.1515 | 282.4516 |
| Seeds boll^-1^ | SEB | 811.4559 | 807.9971 |
| Seeds m^-^² | SM2 | -0.48132 | -0.47901 |
| Thousand-seed weight (g) | TSW | -61.7207 | -60.6249 |
| Bolls m^-^² | BM2 | 3.97766 | 3.96557 |
| Lodging | LOD | 1777 | 1778 |
| Days to flowering | DTF | 1036 | 1037 |
| Days to maturity | DTM | -723.374 | -723.124 |
| Plant height (cm) | PLH | -44.1271 | -44.6004 |
| Branching score | BSC | 512.2274 | 513.2364 |
| Straw weight (g) | PRO | 48.95146 | 48.72011 |
| Fibre (%) | OIL | 421713 | 421721 |
| Lignin (%) | IOD | 239051 | 238992 |
| Shive (%) | PAL | 383264 | 383280 |
| Cell walls (%) | STE | 3619 | 3619 |
| Cellulose (%) | OLE | -2618 | -2617 |
| Protein content (%) | LIO | -396.894 | -397.136 |
| Oil content (%) | LIN | 28.34612 | 28.73649 |
| Iodine value | STR | 59347 | 59350 |
| Linolenic (%) | FIB | 52123 | 52128 |
| Stearic (%) | LIG | 154260 | 154269 |
| Linoleic (%) | SHI | -303.599 | -301.067 |
| Palmitic (%) | CEW | 154427 | 154440 |
| Oleic (%) | CEL | 104027 | 104035 |
| Pasmo score | PAS | 464.7754 | 466.4673 |
| Powdery mildew score | MIL | 240.7311 | 240.7638 |
| Fusarium wilt score | WIL | -150.295 | -149.94 |
