# Supplementary material for: Genetic Variability of 27 Traits in a Core Collection of Flax (Linum usitatissimum L.)
Source: Front Plant Sci. 2017 Sep 21;8:1636. doi: 10.3389/fpls.2017.01636 (PMC5622609; doi:10.3389/fpls.2017.01636)
Supplement: Supplementary file 6 [file Table6.DOCX]

**TABLE S6** Comparison of phenotypic variability of all 27 traits between linseed and fibre types.

| **Trait** | **Abbreviation** | **Linseed** | | |  | **Fibre** | | | ***P*** |
| --- | --- | --- | --- | --- | --- | --- | --- | --- | --- |
|  |  | $\bar{\boldsymbol{x}}$ **± *s*** | **Range** | ***CV*** |  | $\bar{\boldsymbol{x}}$ **± *s*** | **Range** | ***CV*** |  |
| Seed yield (t·ha^-1^) | YLD | 0.80 ± 0.29 | 0.07 ~ 1.46 | 36.64 |  | 0.78 ± 0.18 | 0.44 ~ 1.27 | 23.65 | 0.2580 |
| Seeds boll^-1^ | SEB | 6.10 ± 0.90 | 2.80 ~ 8.12 | 14.81 |  | 6.13 ± 0.82 | 4.50 ~ 7.95 | 13.40 | 0.8767 |
| Seeds m^-2^ | SM2 | 10,944.45 ± 3,162.56 | 2,638.4 ~ 27,714.45 | 28.90 |  | 10,354.58 ± 2,569.96 | 5,246.65 ~ 17,986.43 | 24.82 | 0.0317 |
| Thousand-seed weight (g) | TSW | 5.49 ± 0.89 | 2.9 ~ 8.42 | 16.15 |  | 4.92 ± 0.57 | 3.51 ~ 6.77 | 11.64 | < 0.0001 |
| Bolls m^-2^ | BM2 | 1,788.07 ± 408.29 | 736.32 ~ 3,821.00 | 22.83 |  | 1,683.46 ± 293.96 | 1,082.87 ~ 2,478.45 | 17.46 | 0.0024 |
| Lodging | LOD | 1.4 ± 0.42 | 0.87 ~ 3.32 | 30.25 |  | 1.24 ± 0.27 | 0.90 ~ 2.57 | 21.54 | < 0.0001 |
| Days to flowering | DTF | 50.78 ± 3.03 | 45.53 ~ 70.74 | 5.96 |  | 52.27 ± 2.92 | 47.91 ~ 60.69 | 5.59 | < 0.0001 |
| Days to maturity | DTM | 97.68 ± 3.78 | 89.06 ~ 110.70 | 3.87 |  | 96.49 ± 3.96 | 88.06 ~ 106.57 | 4.11 | 0.0166 |
| Plant height (cm) | PLH | 46.74 ± 9.82 | 23.00 ~ 84.37 | 21.02 |  | 65.5 ± 10.73 | 43.44 ~ 95.49 | 16.39 | < 0.0001 |
| Branching score | BSC | 3.3 ± 0.73 | 1.50 ~ 5.33 | 22.26 |  | 4.15 ± 0.66 | 2.33 ~ 6.00 | 15.90 | < 0.0001 |
| Protein content (%) | PRO | 26.34 ± 1.54 | 16.73 ~ 31.30 | 5.86 |  | 28.62 ± 1.46 | 24.41 ~ 31.01 | 5.11 | < 0.0001 |
| Oil content (%) | OIL | 42.54 ± 1.81 | 37.22 ~ 50.59 | 4.24 |  | 40.79 ± 1.43 | 38.59 ~ 46.97 | 3.51 | < 0.0001 |
| Iodine value | IOD | 185.66 ± 7.58 | 145.41 ~ 202.85 | 4.08 |  | 187.52 ± 4.54 | 165.99 ~ 197.45 | 2.42 | 0.0065 |
| Palmitic (%) | PAL | 5.64 ± 0.55 | 3.34 ~ 8.45 | 9.75 |  | 4.97 ± 0.47 | 3.30 ~ 6.10 | 9.45 | < 0.0001 |
| Stearic (%) | STE | 4.38 ± 1.02 | 2.33 ~ 9.40 | 23.39 |  | 4.16 ± 0.95 | 2.69 ~ 7.87 | 22.94 | 0.0930 |
| Oleic (%) | OLE | 21.3 ± 3.23 | 13.82 ~ 37.97 | 15.18 |  | 20.92 ± 2.26 | 16.15 ~ 31.57 | 10.81 | 0.2124 |
| Linoleic (%) | LIO | 13.92 ± 4.64 | 6.84 ~ 68.43 | 33.35 |  | 15.2 ± 1.41 | 11.61 ~ 19.12 | 9.27 | < 0.0001 |
| Linolenic (%) | LIN | 54.76 ± 5.22 | 5.02 ~ 66.07 | 9.54 |  | 54.72 ± 2.32 | 44.41 ~ 59.69 | 4.24 | 0.7878 |
| Straw weight (g) | STR | 19.86 ± 8.83 | 4.58 ~ 53.09 | 44.46 |  | 36.6 ± 14.27 | 13.96 ~ 75.01 | 38.98 | < 0.0001 |
| Fibre (%) | FIB | 38.13 ± 1.64 | 34.47 ~ 45.15 | 4.29 |  | 39.69 ± 2.51 | 35.89 ~ 46.52 | 6.33 | < 0.0001 |
| Lignin (%) | LIG | 9.54 ± 0.24 | 8.52 ~ 10.08 | 2.48 |  | 9.31 ± 0.36 | 8.32 ~ 9.85 | 3.90 | < 0.0001 |
| Shive (%) | SHI | 62.45 ± 1.65 | 55.38 ~ 66.11 | 2.65 |  | 60.88 ± 2.53 | 53.98 ~ 64.69 | 4.16 | < 0.0001 |
| Cell walls (%) | CEW | 79.01 ± 0.98 | 75.86 ~ 82.01 | 1.24 |  | 79.68 ± 0.67 | 78.29 ~ 81.42 | 0.84 | < 0.0001 |
| Cellulose (%) | CEL | 59.83 ± 2.00 | 54.21 ~ 65.14 | 3.34 |  | 61.13 ± 1.60 | 58.00 ~ 65.21 | 2.61 | < 0.0001 |
| Pasmo score | PAS | 3.63 ± 0.91 | 1.35 ~ 7.15 | 25.11 |  | 2.67 ± 0.70 | 1.63 ~ 4.73 | 26.18 | < 0.0001 |
| Powdery mildew score | MIL | 3.88 ± 1.34 | 1.36 ~ 8.45 | 34.47 |  | 4.94 ± 1.54 | 2.27 ~ 7.54 | 31.19 | < 0.0001 |
| Fusarium wilt score | WIL | 6.82 ± 1.18 | 3.53 ~ 8.96 | 17.24 |  | 6.26 ± 1.06 | 3.91 ~ 8.96 | 16.89 | < 0.0001 |

$\bar{x}$, population mean of linseed or fibre subsets; *s*, standard deviation; *CV*, coefficient of variation; *P*: *P* value for *t* test between linseed and fibre types.

The numbers of accessions in linseed and fibre types are 299 and 92, respectively.
