# Supplementary material for: Genetic Variability of 27 Traits in a Core Collection of Flax (Linum usitatissimum L.)
Source: Front Plant Sci. 2017 Sep 21;8:1636. doi: 10.3389/fpls.2017.01636 (PMC5622609; doi:10.3389/fpls.2017.01636)
Supplement: Supplementary file 7 [file Table7.DOCX]

**TABLE S7** Euclidean distances within and between the four geographical regions in fibre accessions.

|  | **No. of accessions** | **NA** | **WE** | **CEE** | **EA** | **Mean ± *s*** |
| --- | --- | --- | --- | --- | --- | --- |
| North America (NA) | 13 | 24.40 | 27.08 | 26.46 | 45.03 | 32.86 ± 10.55 |
| Western Europe (WE) | 22 |  | 24.66 | 24.23 | 33.85 | 28.39 ± 4.94 |
| Central and Eastern Europe (CEE) | 39 |  |  | 23.96 | 33.12 | 27.94 ± 4.63 |
| Eastern Asia (EA) | 8 |  |  |  | 21.90 | 37.33 ± 6.67 |

The diagonal and upper-right triangle values represent distances within and between geographical regions, respectively. Mean: average distance of a region with all other regions.
