# Supplementary material for: Genetic Variability of 27 Traits in a Core Collection of Flax (Linum usitatissimum L.)
Source: Front Plant Sci. 2017 Sep 21;8:1636. doi: 10.3389/fpls.2017.01636 (PMC5622609; doi:10.3389/fpls.2017.01636)
Supplement: Supplementary file 8 [file Table8.DOCX]

**TABLE S8** Euclidean distances within and between the nine geographical regions in linseed accessions.

|  | **No. of accessions** | **NA** | **SA** | **NE** | **WE** | **CEE** | **SE** | **WA** | **SAS** | **AF** | **Mean ± *s*** |
| --- | --- | --- | --- | --- | --- | --- | --- | --- | --- | --- | --- |
| North America (NA) | 119 | 33.87 | 28.73 | 36.19 | 35.65 | 35.67 | 26.98 | 35.79 | 44.76 | 36.33 | 35.01 ± 5.39 |
| South America (SA) | 18 |  | 20.77 | 28.15 | 25.66 | 27.29 | 17.12 | 27.08 | 36.60 | 27.85 | 27.31 ± 5.29 |
| Northern Europe (NE) | 7 |  |  | 38.25 | 30.79 | 33.58 | 26.62 | 33.16 | 38.82 | 32.66 | 32.50 ± 3.99 |
| Western Europe (WE) | 25 |  |  |  | 25.92 | 30.24 | 24.60 | 29.17 | 32.32 | 28.72 | 29.64 ± 3.53 |
| Central and Eastern Europe (CEE) | 46 |  |  |  |  | 33.22 | 25.59 | 32.99 | 39.41 | 32.74 | 32.19 ± 4.45 |
| Southern Europe (SE) | 5 |  |  |  |  |  | 14.41 | 25.69 | 36.43 | 26.46 | 26.19 ± 5.23 |
| Western Asia (WA) | 14 |  |  |  |  |  |  | 33.39 | 36.94 | 32.02 | 31.61 ± 4.00 |
| Southern Asia (SAS) | 52 |  |  |  |  |  |  |  | 36.21 | 36.05 | 37.67 ± 3.57 |
| Africa (AF) | 8 |  |  |  |  |  |  |  |  | 35.27 | 31.60 ± 3.66 |

The diagonal and upper-right triangle values represent distances within and between geographical regions, respectively. Mean: average distance of a region with all other regions.
