# Supplementary material for: Genetic Variability of 27 Traits in a Core Collection of Flax (Linum usitatissimum L.)
Source: Front Plant Sci. 2017 Sep 21;8:1636. doi: 10.3389/fpls.2017.01636 (PMC5622609; doi:10.3389/fpls.2017.01636)
Supplement: Supplementary file 9 [file Table9.DOCX]

**TABLE S9** Means and standard deviation ($\bar{x}$ ± *s*) of traits for the three clusters obtained from the cluster analysis of the 92 fibre accessions.

| **Trait** | **Cluster (No. of accessions)** | | | |
| --- | --- | --- | --- | --- |
|  | **1 (32)** | | **2 (17)** | **3 (43)** |
| Seed yield (t·ha^-1^) | 0.91 ± 0.19a | 0.65 ± 0.14b | | 0.73 ± 0.13b |
| Seeds boll^-1^ | 6.5 ± 0.71a | 5.13 ± 0.34b | | 6.24 ± 0.72a |
| Seeds m^-2^ | 11,994.47 ± 2,724.57a | 7,714.64 ± 1,438.05c | | 10,177.9 ± 1,772.42b |
| Thousand-seed weight (g) | 4.78 ± 0.69b | 5.49 ± 0.50a | | 4.8 ± 0.32b |
| Bolls m^-2^ | 1,843.55 ± 309.72a | 1,491.07 ± 246.99c | | 1,640.38 ± 238.28b |
| Lodging | 1.29 ± 0.32ab | 1.35 ± 0.30a | | 1.15 ± 0.17b |
| Days to flowering | 50.11 ± 1.36c | 56.71 ± 1.80a | | 52.13 ± 2.08b |
| Days to maturity | 94.43 ± 2.99b | 102.25 ± 2.55a | | 95.76 ± 2.78b |
| Plant height (cm) | 55.27 ± 5.66c | 75.61 ± 12.78a | | 69.11 ± 5.41b |
| Branching score | 3.6 ± 0.48c | 4.67 ± 0.44a | | 4.36 ± 0.57b |
| Protein content (%) | 27.83 ± 1.63b | 28.48 ± 1.22b | | 29.28 ± 1.08a |
| Oil content (%) | 41.35 ± 1.75a | 41.11 ± 1.26a | | 40.25 ± 1.00b |
| Iodine value | 189.53 ± 5.48a | 186.01 ± 4.48b | | 186.63 ± 3.18b |
| Palmitic (%) | 5.12 ± 0.59a | 5.12 ± 0.34a | | 4.79 ± 0.34b |
| Stearic (%) | 3.43 ± 0.51c | 4.95 ± 1.23a | | 4.39 ± 0.71b |
| Oleic (%) | 20.66 ± 2.84a | 20.82 ± 1.75a | | 21.16 ± 1.97a |
| Linoleic (%) | 15.15 ± 1.22a | 14.32 ± 1.22b | | 15.58 ± 1.48a |
| Linolenic (%) | 55.66 ± 2.69a | 54.64 ± 2.37ab | | 54.06 ± 1.75b |
| Straw weight (g) | 23.15 ± 6.40c | 49.94 ± 13.78a | | 41.34 ± 10.28b |
| Fibre (%) | 38.26 ± 1.27b | 38.49 ± 1.38b | | 41.22 ± 2.68a |
| Lignin (%) | 9.52 ± 0.18a | 9.49 ± 0.20a | | 9.09 ± 0.39b |
| Shive (%) | 62.32 ± 1.27a | 62.08 ± 1.38a | | 59.32 ± 2.70b |
| Cell walls (%) | 79.52 ± 0.53a | 79.77 ± 0.57a | | 79.77 ± 0.77a |
| Cellulose (%) | 60.29 ± 1.52b | 61.18 ± 1.24a | | 61.73 ± 1.52a |
| Pasmo score | 3.39 ± 0.59a | 2.13 ± 0.31b | | 2.35 ± 0.38b |
| Powdery mildew score | 4.68 ± 1.53b | 3.58 ± 1.18c | | 5.67 ± 1.24a |
| Fusarium wilt score | 6.88 ± 0.88a | 6.08 ± 1.23b | | 5.86 ± 0.89b |
