# Supplementary material for: Genetic Variability of 27 Traits in a Core Collection of Flax (Linum usitatissimum L.)
Source: Front Plant Sci. 2017 Sep 21;8:1636. doi: 10.3389/fpls.2017.01636 (PMC5622609; doi:10.3389/fpls.2017.01636)
Supplement: Supplementary file 10 [file Table10.DOCX]

**TABLE S10** Means and standard deviation ($\bar{x}$ ± *s*) of traits for the eight clusters obtained from the cluster analysis of the 299 linseed accessions.

| **Trait** | **Cluster (No. of accessions)** | | | | | | | |
| --- | --- | --- | --- | --- | --- | --- | --- | --- |
|  | **1 (12)** | **2 (51)** | **3 (6)** | **4 (80)** | **5 (2)** | **6 (91)** | **7 (10)** | **8 (47)** |
| Seed yield (t·ha^-1^) | 0.64 ± 0.21b | 0.82 ± 0.20b | 0.70 ± 0.09b | 1.10 ± 0.18a | 1.00 ± 0.11a | 0.79 ± 0.19b | 0.34 ± 0.17c | 0.43 ± 0.15c |
| Seeds boll^-1^ | 4.38 ± 0.80f | 6.55 ± 0.65bc | 6.27 ± 0.86bcd | 6.60 ± 0.79b | 7.74 ± 0.15a | 5.84 ± 0.71cde | 5.27 ± 0.91e | 5.79 ± 0.64de |
| Seeds m^-2^ | 9,991.03 ± 3,021.38b | 12,694.65 ± 3,005.44a | 9,992.82 ± 893.31b | 13,352.65 ± 2,208.92a | 14,320.35 ± 1,068.97a | 9,843.96 ± 2,220.09b | 5,233.62 ± 1,373.31b | 8,483.07 ± 2,095.52c |
| Thousand-seed weight (g) | 4.33 ± 0.72d | 4.81 ± 0.56cd | 4.76 ± 0.13cd | 5.44 ± 0.55bc | 5.50 ± 0.18bc | 6.05 ± 0.94b | 7.13 ± 0.55a | 5.39 ± 0.56bc |
| Bolls m^-2^ | 2,140.46 ± 538.39a | 1,972 ± 419.11ab | 1,617.74 ± 92.09cd | 2,045.4 ± 223.40a | 1,871.47 ± 176.14abc | 1,681.35 ± 302.46bcd | 979.64 ± 152.71e | 1,454.16 ± 287.51d |
| Lodging | 1.59 ± 0.54ab | 1.29 ± 0.37bc | 1.00 ± 0.08c | 1.25 ± 0.33bc | 0.96 ± 0.11c | 1.44 ± 0.45ab | 1.79 ± 0.44a | 1.63 ± 0.37ab |
| Days to flowering | 59.88 ± 5.7a | 49.67 ± 1.83d | 51.98 ± 0.45bc | 52.12 ± 2.32bc | 52.54 ± 1.49b | 50.09 ± 1.63cd | 48.33 ± 0.66d | 49.00 ± 1.24d |
| Days to maturity | 106.19 ± 2.95a | 95.19 ± 3.19cd | 95.09 ± 1.66cd | 98.16 ± 2.83bc | 97.86 ± 0.66bc | 98.88 ± 3.34b | 93.34 ± 2.84d | 96.35 ± 2.49bcd |
| Plant height (cm) | 49.87 ± 5.24bc | 48.00 ± 9.34c | 69.85 ± 3.64a | 54.77 ± 5.28b | 54.41 ± 0.83b | 44.48 ± 5.86c | 32.37 ± 6.22d | 35.08 ± 4.15d |
| Branching score | 3.89 ± 0.62a | 3.16 ± 0.70b | 4.11 ± 0.34a | 3.80 ± 0.57a | 4.17 ± 0.71a | 3.19 ± 0.59b | 3.10 ± 0.47b | 2.53 ± 0.49c |
| Protein content (%) | 25.06 ± 0.91d | 27.49 ± 1.73b | 29.43 ± 0.77a | 26.70 ± 0.99bc | 27.70 ± 1.10b | 25.87 ± 1.17cd | 24.88 ± 0.54d | 25.47 ± 1.53cd |
| Oil content (%) | 41.42 ± 2.25bc | 41.33 ± 2.29bc | 40.53 ± 0.50c | 42.71 ± 1.61ab | 43.27 ± 0.03a | 42.80 ± 1.39ab | 43.80 ± 2.11a | 43.32 ± 1.20a |
| Iodine value | 192.01 ± 6.94a | 193.68 ± 4.62a | 186.23 ± 2.22bc | 189.07 ± 3.58ab | 149.33 ± 5.54e | 181.93 ± 4.93cd | 179.36 ± 2.56d | 179.56 ± 5.61d |
| Palmitic (%) | 5.88 ± 0.26b | 5.27 ± 0.62c | 4.68 ± 0.33d | 5.50 ± 0.46bc | 6.41 ± 0.25a | 5.95 ± 0.52b | 5.95 ± 0.18b | 5.64 ± 0.22bc |
| Stearic (%) | 4.35 ± 0.46cd | 3.55 ± 0.56e | 4.29 ± 0.52cde | 3.69 ± 0.70de | 3.78 ± 0.16de | 4.82 ± 0.78bc | 5.87 ± 0.78a | 5.35 ± 0.90ab |
| Oleic (%) | 18.44 ± 3.20de | 18.52 ± 2.56de | 21.71 ± 1.79bc | 20.30 ± 2.37cd | 16.28 ± 0.63e | 22.39 ± 2.37abc | 22.93 ± 0.95ab | 24.44 ± 3.23a |
| Linoleic (%) | 11.77 ± 0.98e | 13.94 ± 1.77bcd | 15.64 ± 1.40b | 14.50 ± 1.64bc | 64.65 ± 5.35a | 13.75 ± 2.04cd | 12.50 ± 1.43de | 11.72 ± 1.57e |
| Linolenic (%) | 59.73 ± 4.28a | 58.77 ± 3.13a | 53.73 ± 1.32bc | 55.96 ± 1.97b | 9.14 ± 5.83d | 53.09 ± 3.06c | 52.60 ± 1.29c | 52.87 ± 2.80c |
| Straw weight (g) | 32.42 ± 10.09b | 20.22 ± 6.5cd | 39.09 ± 9.01a | 25.64 ± 7.38c | 21.98 ± 5.67cd | 17.38 ± 5.16d | 7.95 ± 2.00e | 11.21 ± 3.40e |
| Fibre (%) | 37.05 ± 1.68d | 37.71 ± 1.05cd | 43.39 ± 1.4a | 38.44 ± 1.44c | 38.52 ± 1.99c | 38.10 ± 1.13cd | 41.31 ± 1.32b | 37.05 ± 1.05d |
| Lignin (%) | 9.69 ± 0.25a | 9.60 ± 0.15ab | 8.78 ± 0.21d | 9.49 ± 0.21b | 9.48 ± 0.28b | 9.54 ± 0.16ab | 9.09 ± 0.19c | 9.69 ± 0.15a |
| Shive (%) | 63.56 ± 1.68a | 62.86 ± 1.06ab | 57.15 ± 1.41d | 62.15 ± 1.45b | 62.06 ± 2.02b | 62.49 ± 1.15ab | 59.21 ± 1.34c | 63.54 ± 1.05a |
| Cell walls (%) | 78.22 ± 1.23c | 79.45 ± 0.75b | 79.64 ± 0.80b | 79.34 ± 0.73b | 78.98 ± 0.88bc | 78.77 ± 0.85bc | 80.61 ± 0.86a | 78.24 ± 0.89c |
| Cellulose (%) | 61.76 ± 2.13ab | 59.35 ± 1.38cd | 62.27 ± 1.62a | 61.42 ± 1.38ab | 60.64 ± 0.07bc | 59.56 ± 1.53cd | 59.14 ± 1.93d | 57.48 ± 1.24e |
| Pasmo score | 2.53 ± 0.95e | 3.50 ± 0.83cd | 2.49 ± 0.22e | 3.06 ± 0.48de | 3.08 ± 0.13de | 3.77 ± 0.66c | 5.40 ± 0.79a | 4.54 ± 0.64b |
| Powdery mildew score | 2.47 ± 1.39d | 4.11 ± 1.30bc | 5.79 ± 1.09a | 3.62 ± 1.22cd | 3.08 ± 0.45cd | 4.14 ± 1.28bc | 5.19 ± 1.78ab | 3.51 ± 1.03cd |
| Fusarium wilt score | 7.36 ± 1.22ab | 6.73 ± 1.10b | 5.61 ± 1.05c | 5.81 ± 0.98c | 7.21 ± 0.51ab | 7.18 ± 0.86ab | 8.12 ± 0.37a | 7.77 ± 0.66a |

Different letters between clusters for a trait indicate significant difference at the 0.05 probability level.
